# DDR1 as a key prognostic biomarker in non-small cell lung cancer: identification, validation, and potential therapeutic implications

**DOI:** 10.3389/fimmu.2025.1690829

**Published:** 2025-11-28

**Authors:** RenCai Lu, Lu Qian, XueQin Sun, JianFang Zhang, YeQian Cui, HuiMei Su, DongDong Xv, ShaoBo Wang

**Affiliations:** 1 Faculty of Life Science and Technology, Kunming University of Science and Technology, Kunming, China; 2 School of Medicine, Kunming University of Science and Technology, Kunming, China; 3 Department of Nuclear Medicine, The First People’s Hospital of Yunnan Province, The Affiliated Hospital of Kunming University of Science and Technology, Kunming, Yunnan, China; 4 Department of Pathology, The First People’s Hospital of Yunnan Province, The Affiliated Hospital of Kunming University of Science and Technology, Kunming, Yunnan, China; 5 Department of Nuclear Medicine, No.926 Hospital, Joint Logistics Support Force of PLA, Kaiyuan, Yunnan, China

**Keywords:** discoidin domain receptor 1, non-small cell lung cancer, bioinformatics, machine learning, immunotherapy

## Abstract

**Background:**

Non-small cell lung cancer (NSCLC) remains the leading cause of cancer-related death, with a limited response to immune checkpoint inhibitors (ICIs). Discoidin domain receptor 1 (DDR1) is a collagen-binding kinase that is implicated in tumor progression and immune escape, but its role in NSCLC is unclear. This study aimed to clarify the clinical significance and therapeutic potential of DDR1 via bioinformatics, machine learning, *in vitro* experiments, and clinical sample analysis.

**Materials and Methods:**

NSCLC patients were stratified by DDR1 expression based on retrospective RNA-seq data from The Cancer Genome Atlas (TCGA); after quality control, 495 lung adenocarcinoma (LUAD) and 481 lung squamous cell carcinoma (LUSC) tumor samples, together with 57 LUAD and 48 LUSC normal samples, were retained for further analysis. The analyses included survival, mutation, immune landscape, drug sensitivity, single-cell heterogeneity, and functional Gene Ontology (GO) and Kyoto Encyclopedia of Genes and Genomes (KEGG) enrichment analyses, accounting for the heterogeneity among NSCLC subtypes. A machine learning-based 4-gene prognostic model was constructed and externally validated using two independent datasets: GSE30219 (GPL570 platform, 278 NSCLC samples) and GSE41271 (GPL6884 platform, 274 NSCLC samples). The biological functions of DDR1 were further evaluated via *in vitro* assays and immunohistochemistry (IHC) of clinical samples.

**Results:**

High DDR1 expression was correlated with advanced T stage (P<0.05), poor progression-free survival (PFS) (HR = 1.62, P<0.001), and an immunosuppressive microenvironment. Drug sensitivity analysis revealed that high DDR1 expression was associated with reduced sensitivity to methotrexate but increased sensitivity to vinblastine, doxorubicin, cisplatin, and docetaxel, with no significant difference observed for gefitinib. Single-cell heterogeneity analysis revealed that DDR1 was enriched in tumor-associated macrophages and neutrophils. A LASSO and random survival forest (RSF) machine learning algorithm revealed a 4-gene signature (PKP2, DKK1, TEF, and GJB5) with strong prognostic value (C-index=0.728). DDR1 knockdown suppressed cell proliferation, migration, and invasion and induced apoptosis in NSCLC cell lines. IHC of clinical samples confirmed DDR1 overexpression in 55.88% of NSCLC patients.

**Conclusion:**

Our study demonstrated that DDR1 promotes tumor progression and immune evasion and is frequently overexpressed in NSCLC patients, suggesting that DDR1 is a potential prognostic biomarker and therapeutic target.

## 1 Introduction

Lung cancer incidence has risen from 11.4% to 12.4% of all cancers worldwide between 2020 and 2022, with its mortality increasing from 18.0% to 18.7% of global cancer deaths, making it the most frequently diagnosed malignancy. Non-small cell lung cancer (NSCLC) accounts for approximately 85% of all cases (1–3). The majority of NSCLC patients are diagnosed at an advanced or even metastatic stage, with a five-year survival rate of less than 15% (4, 5). Despite advances in conventional, targeted, and immune checkpoint therapies, patient outcomes remain variable due to individual differences and tumor escape mechanisms (4, 6, 7). Currently, the most widely employed immunotherapy strategy in clinical practice is PD-1 and CTLA-4 immune checkpoint inhibition. However, nearly half of patients with NSCLC do not respond to ICIs, and some patients with high expression levels fail to respond to monotherapy (8). Despite significant advances in immunotherapy, available clinical biomarkers remain suboptimal, as none possess sufficient sensitivity or specificity to reliably predict responders or non-responders (8). The shortcomings associated with these treatments underscore the urgent need for reliable prognostic biomarkers to guide personalized treatment strategies.

Discoidin domain receptor 1 (DDR1), a collagen-binding receptor tyrosine kinase encoded on chromosome 6p21.3, promotes tumor progression and immune exclusion by regulating cell motility, extracellular matrix remodeling, and collagen alignment that restricts immune cell infiltration (9, 10). Aberrant DDR1 activation has been implicated in multiple cancers, including pancreatic, cervical, hepatic, and breast malignancies (11–16). In a triple-negative breast cancer mouse model, DDR1 knockout enhanced CD8^+^ and CD4^+^ T-cell infiltration, increased IFN-γ production, disrupted peripheral collagen organization, and suppressed tumor growth (10). However, another study reported that pharmacological inhibition or genetic deletion of DDR1 increased tumor burden and inadvertently promoted a protumorigenic microenvironment (17). These findings suggest that while DDR1 is a potential therapeutic target, its role in shaping the tumor microenvironment (TME) and clinical implications in NSCLC remain unclear and require further investigation. Unlike conventional biomarkers such as PD-L1 with limited predictive accuracy, DDR1 may provide complementary or enhanced predictive value through its dual role in extracellular matrix remodeling and immune exclusion.

To address these gaps, we employed integrative bioinformatics approaches to comprehensively characterize DDR1 expression patterns, genetic alterations, immune landscape associations, and prognostic significance in NSCLC, supported by machine learning and external validation (18).

In this study, we identified DDR1 as a key oncogenic driver in NSCLC that modulates the immune microenvironment and influences immunotherapy response. Using machine learning, we developed and externally validated a robust prognostic model for clinical outcome prediction. DDR1 expression was further confirmed in clinical samples, and *in vitro* experiments demonstrated that DDR1 knockdown influence NSCLC cell function. These findings establish DDR1 as a key oncogenic regulator in NSCLC progression and offer new insights into therapeutic strategies targeting DDR1.

## 2 Materials and methods

### 2.1 Data acquisition and preprocessing

RNA-seq data for TCGA-LUAD and TCGA-LUSC cohorts were obtained from the TCGA website (https://portal.gdc.cancer.gov/) and converted to TPM, then log2(TPM + 1) transformed. After excluding samples with missing follow-up or incomplete clinical and staging information, 495 LUAD tumor and 57 control samples, and 481 LUSC tumor and 48 control samples were retained. These were combined as NSCLC data for further analysis. NSCLC datasets with follow-up data, GSE30219 (19) and GSE41271 (20), were downloaded from GEO (https://www.ncbi.nlm.nih.gov/geo/) via GEOquery (https://bioconductor.org/packages/release/bioc/html/GEOquery.html, v2.70.0) (21). GSE30219 (GPL570 platform) includes 278 NSCLC samples, and GSE41271 (GPL6884 platform) includes 274, both used for external validation.

DNA methylation data for TCGA-LUAD and TCGA-LUSC were downloaded from UCSC-Xena (https://toil.xenahubs.net) using HumanMethylation450 BeadChip, including 732 NSCLC tumor samples. DDR1 methylation was calculated as the mean beta value across all sites (22). Somatic mutation data were obtained using TCGAbiolinks (https://bioconductor.org/packages/release/bioc/html/TCGAbiolinks.html, v2.30.0) (23) and visualized with maftools (https://bioconductor.org/packages/release/bioc/html/maftools.html, v2.18.0) (24). CNV analysis used “Masked Copy Number Segment” data via TCGAbiolinks.

scRNA-seq data (GSE117570 (25)) were downloaded from GEO, including eight samples (GSM3304007–GSM3304014). Low-quality cells were removed by filtering out those with fewer than 200 detected genes or with mitochondrial gene expression exceeding 5% of total reads to ensure data reliability (26–28), leaving 11,481 cells for analysis.

### 2.2 Survival analysis

The surv_cutpoint function from the survminer package was utilized to determine the optimal cutoff value for DDR1 expression in NSCLC patients, with survival time and survival status used as outcome variables (29). On the basis of this optimal cutoff, NSCLC patients were divided into high and low DDR1 expression groups. Kaplan–Meier analysis was then performed using the survival (https://cran.r-project.org/web/packages/survival/index.html, version = 3.4-8) and survminer packages to investigate the correlation between patient survival time and NSCLC.

### 2.3 Construction of a nomogram based on DDR1 expression for NSCLC prognosis prediction

Univariate and multivariate Cox regression analyses were performed on DDR1 expression and clinical factors. On the basis of these results, a nomogram (30) was constructed using the rms package (https://cran.r-project.org/web/packages/rms/, version = 6.8-0) (31). Decision curve analysis (DCA) is a straightforward method for evaluating the clinical utility of prediction models, diagnostic tests, and molecular biomarkers. Therefore, calibration curves and DCA plots for 1-year, 3-year, and 5-year predictions were drawn to assess the predictive accuracy of the model.

### 2.4 Cellular mutations and copy number variations

The maftools package in R was used to visualize the somatic mutation profiles between the DDR1 high-expression and low-expression groups in NSCLC, and the mutation differences between the two groups were compared. The results are displayed in a waterfall plot. Additionally, GISTIC 2.0 analysis (32) was conducted on the downloaded CNV segments via GenePattern (https://cloud.genepattern.org) (33) to study the differences in copy number variations between the DDR1 high-expression and low-expression groups.

### 2.5 DDR1 and immune-related features

Four algorithms were used to assess immune infiltration in NSCLC tumor samples. CIBERSORT (https://cibersort.stanford.edu/) estimated the proportions of 22 immune cell types using a known reference matrix and support vector regression (34). ESTIMATE (https://bioinformatics.mdanderson.org/estimate/) inferred tumor purity and stromal/immune cell content based on transcriptomic data via the ESTIMATE R package (35), generating ESTIMATEScore, ImmuneScore, StromalScore, and TumorPurity.

MCPcounter (http://github.com/ebecht/MCPcounter) (36) quantified the abundance of nine immune cell types using whole-transcriptome data. TIMER (https://cistrome.shinyapps.io/timer/) (37) provided immune infiltration estimates through its online platform. These tools jointly evaluated immune infiltration in NSCLC, and Pearson correlation analysis assessed the relationship between infiltration and risk score (RS).

Seven immune modulatory gene types were retrieved from previous studies (38) and their correlation with DDR1 expression was analyzed. To evaluate DDR1’s role in immunotherapy response, immune-related signatures were collected, including cancer immune cycle genes (39), cytotoxic activity (CYT), and tertiary lymphoid structure (TLS) markers (40, 41). CYT and TLS scores were calculated via ssGSEA. The TIDE algorithm (http://tide.dfci.harvard.edu) (42) was used to predict immunotherapy response and immune escape. Tumor mutation burden (TMB) data were obtained from TCGA. Wilcoxon tests compared scores between high and low DDR1 expression groups.

### 2.6 Drug sensitivity prediction

To assess the sensitivity of NSCLC patients to common chemotherapy drugs, the Cancer Drug Sensitivity Genomics database (https://www.cancerrxgene.org/) (43) was used to estimate the sensitivity of each patient to NSCLC chemotherapy drugs. The pRRophetic package in R was then used to calculate the half-maximal inhibitory concentration (IC50), with IC50 values z-score normalized across cell lines to account for baseline variability (44). The Wilcoxon test was applied to compare differences in drug sensitivity between the high and low DDR1 expression groups.

### 2.7 Cell population annotation

The Seurat object for single-cell data was visualized using UMAP, revealing 16 clusters. Through manual annotation on the basis of cell type marker genes, 13 distinct cell types were identified on the basis of their markers as follows: B cells (IGHG1, IGHA1, and IGKC), CD8 T cells (CD8B and CD8A), cytotoxic cells (KLRF1, CTSW, and KLRB), dendritic cells (CLEC10A and MS4A6A), fibroblasts (PROCR and CD151), M1 macrophages (CCR7), M2 macrophages (MRC1, CD163, PPARG, and TREM2), macrophages (GPC4, RAI14, and BCAT1), monocytes (VCAN, FCN1, and IL1B), neutrophils (VNN3 and SLC22A4), NK cells (PSMD4 and TINAGL1), other T cells (TRAT1, CD96, and ITM2A), and T helper cells (BATF, ANP32B, and SNRPD1). Differential gene expression between these cell types was assessed using the FindAllMarkers function, and the results were visualized via a heatmap.

### 2.8 Pseudotime analysis

Pseudotime analysis allows for the ordering of cells along a trajectory on the basis of their gene expression profiles, effectively mapping each cell to a corresponding position in the developmental trajectory. By analyzing gene expression status, cells can be grouped into multiple differentiation states, and an intuitive lineage tree of the predicted differentiation and developmental trajectories of the cells can be generated (45). The results of pseudotime analysis require confirmation of the differentiation starting and ending points on the basis of the trajectory distribution of cell types and the changes in the expression of characteristic genes. For this analysis, the monocle package in R (https://bioconductor.org/packages/release/bioc/html/monocle.html, version = 2.30.0) was used to perform the pseudotime analysis (46).

### 2.9 CellChat analysis

To study intercellular communication and identify the mechanisms of signaling molecules at single-cell resolution, the CellChat package in R (https://github.com/sqjin/CellChat, version = 1.6.1) was used for cell communication analysis (47). CellChat is a public knowledge database that contains information on ligands, receptors, cofactors, and their interactions, along with pathway annotations. Using social network analysis tools, pattern recognition methods, and manifold learning techniques, CellChat identifies differentially expressed ligands and receptors in each cell type and clusters various communication patterns across different cell groups and pathways. Through these analyses, specific signaling roles of each cell group can be determined, and novel functional intercellular communication mechanisms for certain cell types can be discovered.

### 2.10 Differentially expressed gene analysis

Differentially expressed genes (DEGs) between samples with high DDR1 expression and low DDR1 expression were analyzed using the limma package (https://bioconductor.org/packages/release/bioc/html/limma.html, version = 3.58.1) (48). Genes with |logFC| > 0.5 and P value < 0.05 were selected as DEGs for further investigation (49).

### 2.11 Enrichment analysis

To explore functional and pathway differences between the two groups, enrichment analyses were conducted on the DEGs. Gene Ontology (GO) analysis (50) (covering BP, MF, and CC categories) and Kyoto Encyclopedia of Genes and Genomes (KEGG) pathway analysis (51) were performed using the clusterProfiler package in R (52), with false discovery rate (FDR) < 0.05 indicating significance.

Gene set enrichment analysis (GSEA) (53), implemented via clusterProfiler, assessed biological process differences between the groups, with FDR < 0.05 as the threshold. Gene set variation analysis (GSVA) (54), a nonparametric method for evaluating gene set enrichment across samples, was conducted using the GSVA package in R.

The “h.all.v7.5.2.symbols.gmt” gene set from MSigDB (55) was used for GSVA on high and low DDR1 expression groups. Limma was applied to compare pathway scores (P.adjust < 0.05, |logFC| > 0.1), and results were visualized with a heatmap (56).

### 2.12 Construction of a prognostic model for NSCLC using machine learning algorithms

Univariate Cox analysis was first performed on DEGs in both training and validation datasets. Genes with p < 0.05 and consistent hazard ratio directions in at least two datasets were selected as key genes (57). These were used to construct a prognostic model for NSCLC via a multi-step computational framework.

In the TCGA-NSCLC dataset, ten machine learning algorithms were applied: random survival forest (RSF), elastic net (Enet), lasso, ridge, stepwise Cox, Cox boost, plsRcox, SuperPC, GBM, and survival support vector machine (survival-svm) (57, 58). Some algorithms (e.g., lasso, stepwise Cox, RSF) included feature selection. These were combined into 101 model combinations via 10-fold cross-validation, and their performance was evaluated by C-index across TCGA, GSE30219, and GSE41271. The model with the highest average C-index was selected as optimal.

Using this model, prognostic genes were identified and used to calculate a risk score (RS) for each patient. The model’s performance was validated using ROC curves with the timeROC package in R (59). The optimal RS cutoff was determined using surv_cutpoint from survminer (29), classifying patients into high-RS and low-RS groups. Kaplan–Meier analysis (survival and survminer packages) assessed the RS’s prognostic value.

### 2.13 Validation of *in vitro* experiments

The human NSCLC cell lines HARA, NCI-H292, and CALU-3 were acquired from the Kunming Institute of Zoology, Chinese Academy of Sciences, and cultured under standard conditions according to the provider’s protocols. Reverse transcription−quantitative (RT–q)PCR, western blotting and immunofluorescence were used to select two cell lines with high DDR1 expression for subsequent experiments. The primers used for RT–qPCR were as follows: for DDR1, forward: CCGACTGGTTCGCTTCTACC, reverse: CGGTGTAAGACAGGAGTCCATC; for β-actin, forward: CATGTACGTTGCTATCCAGGC, reverse: CTCCTTAATGTCACGCACGAT. Log-phase cells were trypsinized, seeded in 24-well plates (1×10^5^/well), and transfected with DDR1-small interfering (si) RNA using DMEM-based complexes. After transfection (48 h), the cells were cultured in supplemented medium, and DDR1 knockdown was confirmed via RT–qPCR and western blotting. The functional assays used were as follows: (1) Cell viability: Absorbance (450 nm) was measured at 0–72 h after CCK-8 (C0037, Beyotime, China) incubation; (2) Colony formation assay: 1×10^3^ transfected cells/well were cultured for 14 days, fixed with formaldehyde, crystal violet-stained for 15 min, and counted (×40 magnification); (3) Migration/Invasion: 1×10^4^ cells in Matrigel-coated (invasion) or uncoated (migration) Transwell chambers were quantified after 24 h via crystal violet staining; (4) Wound healing: Monolayer scratches were imaged before and after 24 h of incubation; (5) Adhesion: Matrigel-coated 96-well assays were analyzed via 0.3% crystal violet staining; and (6) Apoptosis: Annexin V-FITC/PI (Absin, China)-stained cells were assessed by flow cytometry. All experiments included triplicate measurements and statistical validation.

### 2.14 Validation of pathological features in clinical samples

Paraffin-embedded tissue samples obtained from 34 patients (mean ± SD age, 62.58 ± 9.65 years; 7 females, 27 males) were retrospectively subjected to pathological examination. Ethical approval for the study was received from the Institutional Review Board of the First People’s Hospital of Yunnan Province (Approval No. KHLL2025-KY131) and was conducted in accordance with the Declaration of Helsinki and Good Clinical Practice Guidelines. The requirement for informed consent from all patients was waived because of the retrospective nature of the study. Patient tissue samples were cut into 4-μm-thick sections, deparaffinized, and rehydrated before being treated with antigen retrieval solution (10 mmol/L sodium citrate buffer, pH 6.0) and then reacted with anti-DDR1 antibodies (1:3200; Cell Signaling Technology). Immunoreactivity was assessed by one pathologist with 7 years of experience in a blinded fashion as follows: no staining (-); mild staining (+); moderate staining (++); and strong staining (+++). Quantitative immunohistochemical (IHC) scoring was conducted using ImageJ software to compare the staining intensities between NSCLC tumor tissues and paired adjacent normal pulmonary tissues.

### 2.15 Statistical analysis

**Figure 1:**
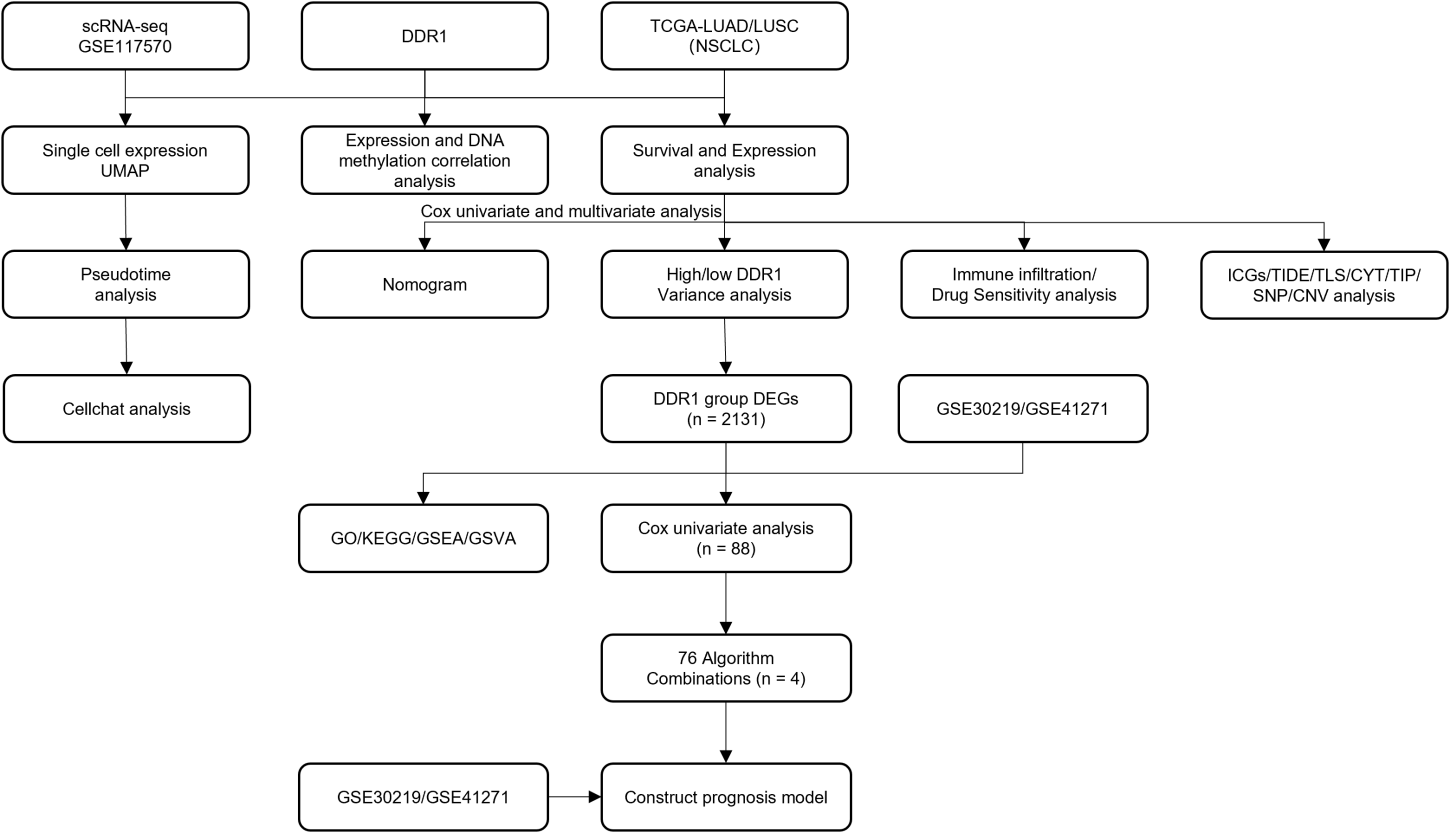
Flowchart of the bioinformatic analysis process.

Flowchart of the bioinformatic analysis process was shown in **Figure 1**. All bioinformatic data calculations and statistical analyses were performed using R programming (https://www.r-project.org/, version 4.1.2). For comparisons of continuous variables between two groups, statistical significance for normally distributed variables was assessed using the independent Student’s t test, whereas the Mann–Whitney U test (i.e., Wilcoxon rank-sum test) was used to analyze differences for nonnormally distributed variables. Differences between the two groups were compared using the R package ggpubr (60), survival analysis was conducted using the survival package in R, and Kaplan–Meier survival curves were generated to display survival differences. The log-rank test was used to assess the significance of survival time differences between the two patient groups, and the results were visualized using the survminer package (61). Spearman’s correlation analysis was used to calculate the correlation coefficients between different molecules. In this study, all the statistical P values were two-sided, with P < 0.05 considered statistically significant. GraphPad Prism (version 9.5.1) and ImageJ (http://imagej.org) were used for the analysis of results of *in vitro* experiments and pathological examination of clinical samples.

## 3 Results

### 3.1 DDR1 expression differences and survival analysis

**Figure 2:**
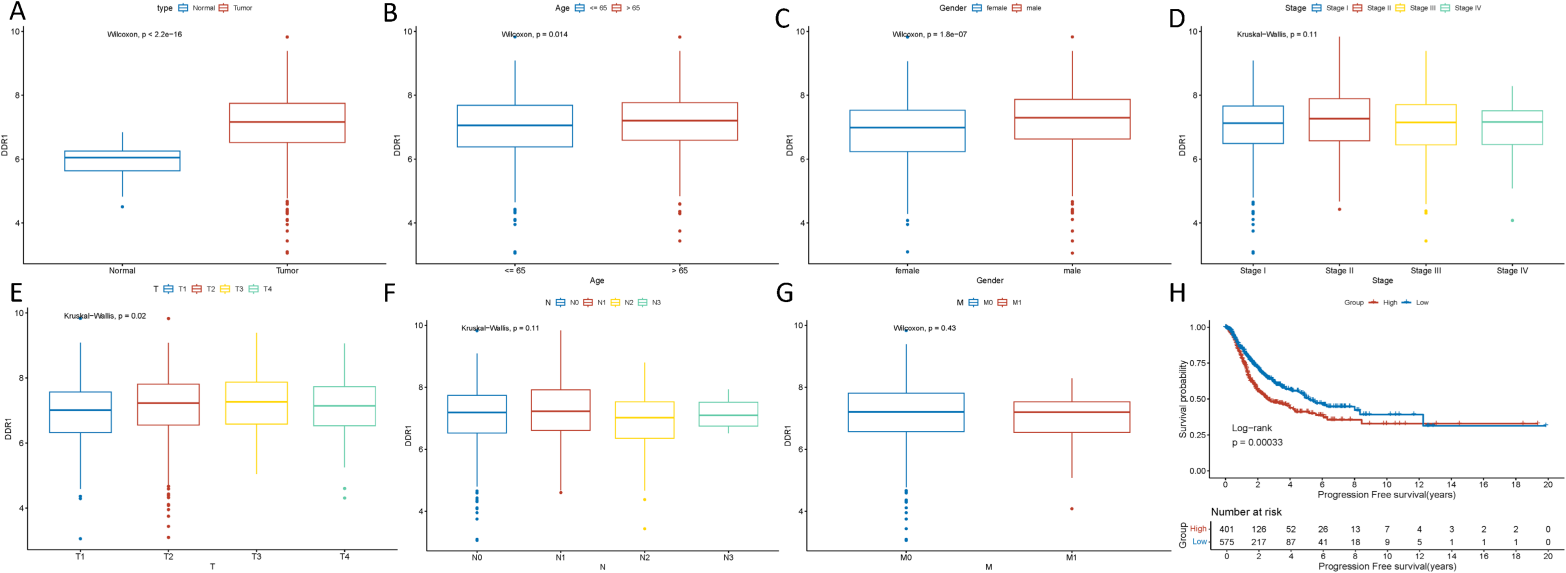
Differential expression of DDR1 and survival analysis in the TCGA dataset. **(A)** Differential expression of DDR1 between tumor and control samples. **(B)** Differential expression of DDR1 between different age groups. **(C)** Differential expression of DDR1 between different sex groups. **(D)** Differential expression of DDR1 across different pathological stages. **(E)** Differential expression of DDR1 across different T stages. **(F)** Differential expression of DDR1 across different N stages. **(G)** Differential expression of DDR1 across different M stages. **(H)** Survival analysis results.

Differential expression analysis of DDR1 in different subgroups of the TCGA dataset revealed that DDR1 expression was significantly higher in tumor samples than in control samples (Tumor: 7.06 ± 0.968 *vs*. Normal 5.95 ± 0.465, P < 0.05) (**Figure 2A**). DDR1 expression was also significantly greater in tumor samples from patients over 65 years of age (over 65 years: 7.14 ± 0.917 *vs*. under 65 years: 6.94 ± 1.03, P < 0.05) and females (female: 6.89 ± 0.95 *vs*. Male: 7.18 ± 0.963, P < 0.05) compared to the corresponding reference groups (**Figures 2B, C**). DDR1 expression was greater in T3 stage samples (T1: 6.93 ± 0.97 *vs*. T2: 7.10 ± 0.965 *vs*. T3: 7.21 ± 0.913 *vs*. T4: 7.01 ± 1.10, P < 0.05) (**Figure 2D**). No statistically significant difference in DDR1 expression was detected across groups with different M stages, N stages, and pathological stages (P > 0.05) (**Figures 2E–G**).

Using the surv_cutpoint function from the survminer package, the optimal cutoff point for DDR1 expression was determined on the basis of the survival time and status of NSCLC patients. This cutoff was then used to classify the patients into high and low DDR1 expression groups (**Supplementary Table 1**). Survival analysis was performed on the basis of these groupings to investigate the correlation between patient survival time and NSCLC features. The analysis results revealed that in the TCGA dataset, the high DDR1 expression group had significantly shorter progression-free survival (PFS) (P < 0.05) (**Figure 2H**).

### 3.2 Nomogram for predicting NSCLC prognosis based on DDR1 expression

**Figure 3:**
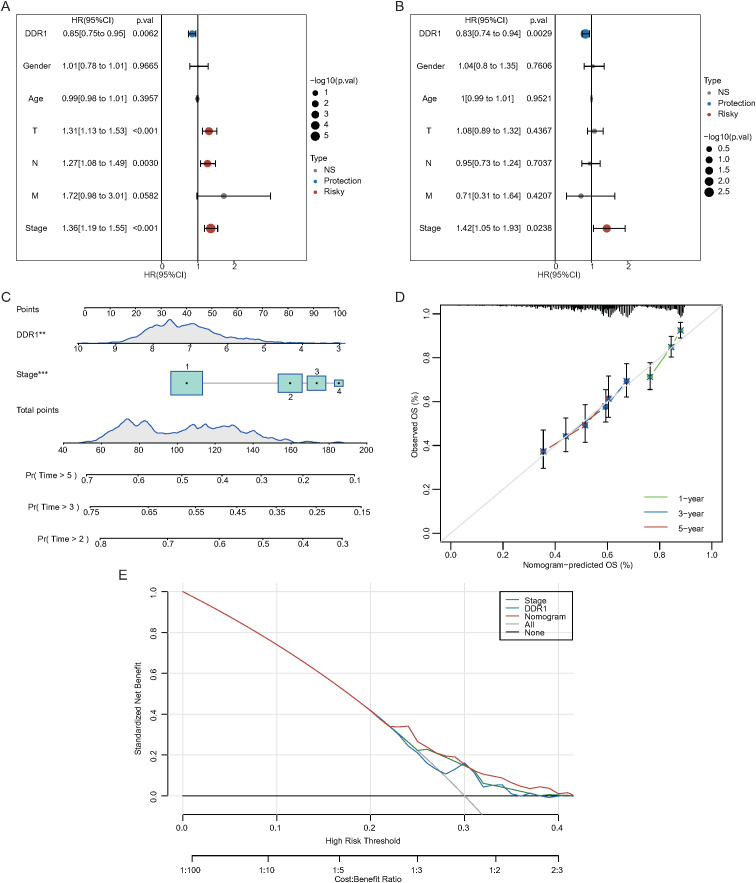
Construction of a nomogram for NSCLC patients based on DDR1 expression from the TCGA database. **(A)** Univariate Cox analysis. **(B)** Multivariate Cox analysis. **(C)** Nomogram for clinical features. **(D)** Calibration curves for 1-, 3-, and 5-year outcomes. **(E)** Decision curve analysis (DCA) for the nomogram model.

Univariate and multivariate risk regression analyses revealed that the T stage (P < 0.05), N stage (P < 0.05), pathological stage (P < 0.05), and DDR1 expression (P < 0.05) were significantly associated with NSCLC prognosis (**Figure 3A**). Multivariate analysis further identified the pathological stage and DDR1 expression as independent prognostic factors (**Figure 3B**). A nomogram based on these factors was constructed (**Figure 3C**), and DCA was used to assess its clinical utility at 1, 3, and 5 years (**Figure 3E**). Calibration curves (**Figure 3D**) revealed that predictions of the 1-year nomogram model were most consistent with those of the ideal model, with other time points also showing high accuracy.

### 3.3 Mutational differences between the high and low DDR1 expression groups

**Figure 4:**
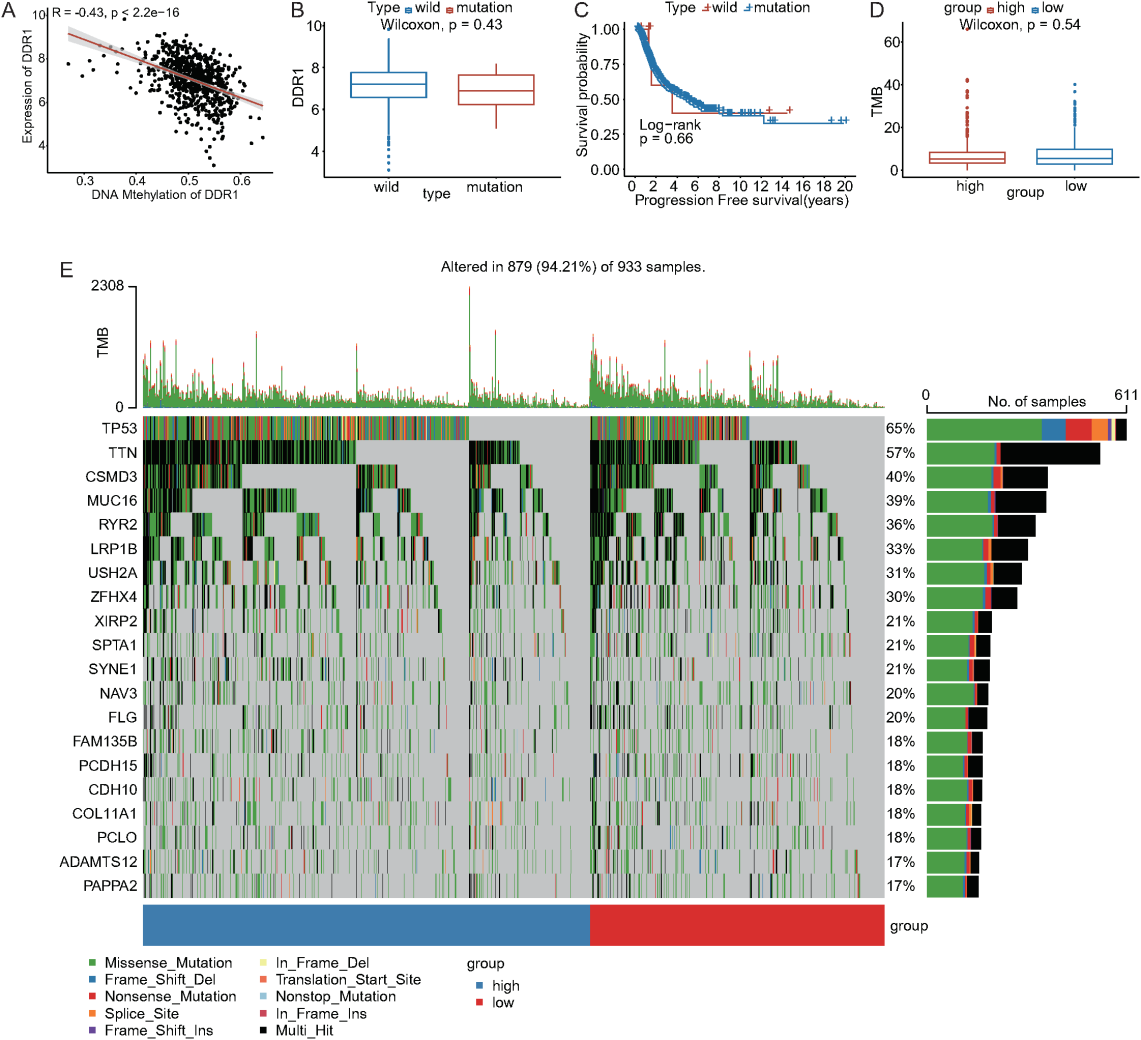
Somatic mutation analysis between the high and low DDR1 expression groups. **(A)** Correlation analysis between DDR1 expression and the DNA methylation level. **(B)** DDR1 expression differences between the DDR1-mutant and wild-type groups. **(C)** Survival analysis between the DDR1-mutant and wild-type groups. **(D)** Distribution of TMB between the high and low DDR1 expression groups. **(E)** Waterfall plot showing common somatic gene mutations, with a bar chart on the right indicating the mutation frequency in each group.

The expression level of DDR1 was significantly negatively correlated with the DNA methylation level (R = -0.43, P < 0.05) (**Figure 4A**). On the basis of DDR1 mutation status, NSCLC samples were categorized into DDR1-mutant and DDR1-wild-type groups. No significant differences in DDR1 expression levels or survival times were detected between the DDR1-mutant and wild-type groups (P > 0.05) (**Figures 4B, C**). Furthermore, no statistically significant differences in the tumor mutational burden (TMB) were found between the high and low DDR1 expression groups (**Figure 4D**).

Compared with common somatic mutations in NSCLC, distinct mutation patterns were observed between the DDR1 expression groups (**Figure 4E**). CNV analysis revealed that in the high DDR1 expression group, amplifications on chromosomes 3, 8, and 11 and deletions on chromosomes 2 and 9 were more frequent (**Supplementary Figures 1A, B**). In the low DDR1 expression group, amplifications on chromosomes 1, 8, and 14 and deletions on chromosome 9 were more common (**Supplementary Figures 1C, D**).

### 3.4 Association between DDR1 and immune-related features

**Figure 5:**
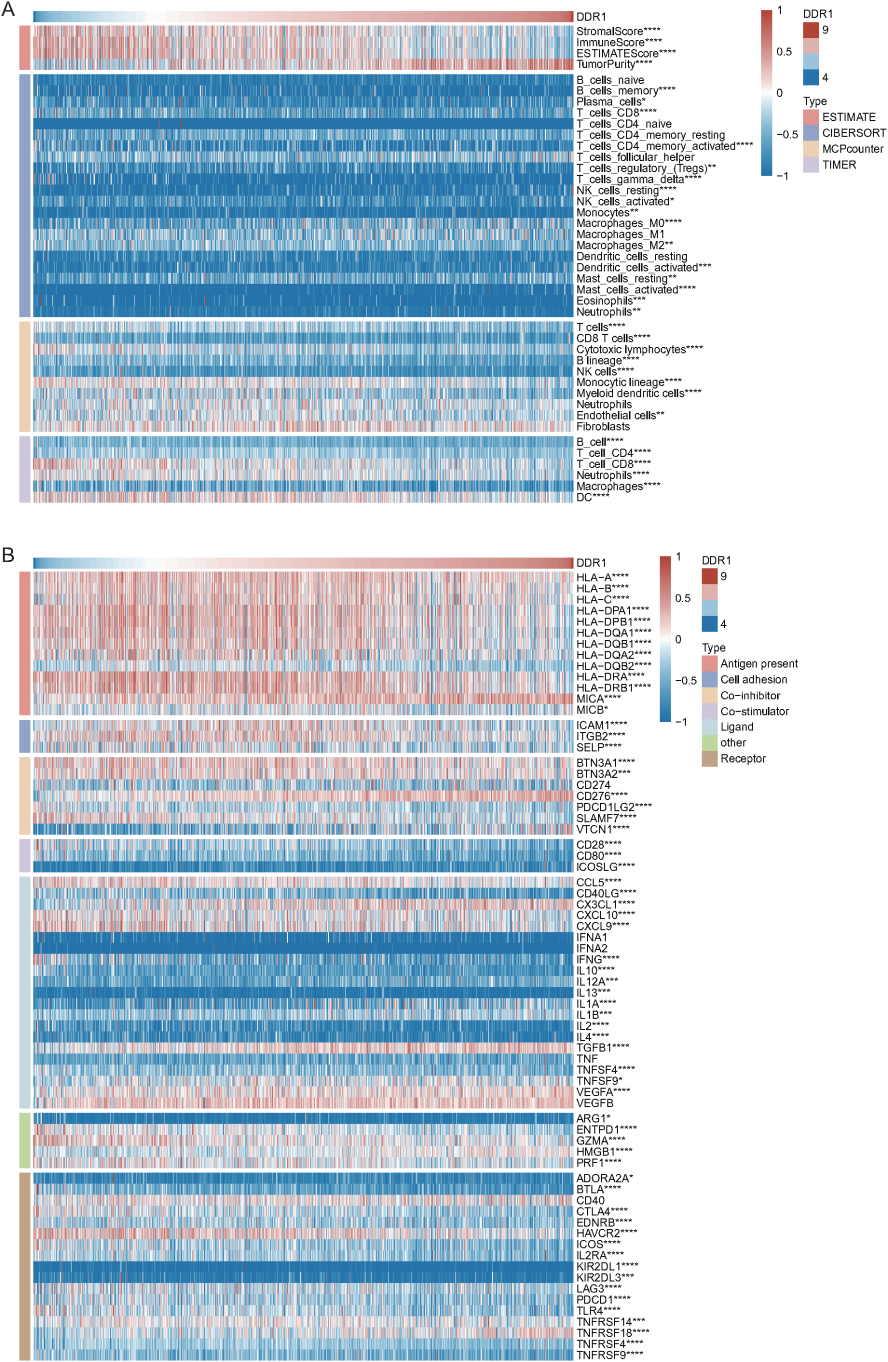
Immune infiltration analysis. **(A)** Correlations between immune cell infiltration and DDR1 expression evaluated by four algorithms. **(B)** Correlations between the expression levels of DDR1 and those of seven types of immunoregulatory genes.

Immune and stromal cell infiltration levels were estimated using four algorithms. Pearson correlation analysis revealed significant associations between DDR1 expression and immune cell infiltration. Among the immune deconvolution algorithms, the strongest correlations were observed for T_cells_CD4_memory_activated (R = -0.18, P < 0.001) and Macrophages_M0 (R = 0.17, P < 0.001) in CIBERSORT (16 cell types); ImmuneScore (R = -0.46, P < 0.001) and ESTIMATEScore (R = -0.45, P < 0.001) in ESTIMATE (4 cell types); T_cells (R = -0.31, P < 0.001) and Monocytic_lineage (R = -0.30, P < 0.001) in MCPcounter (8 cell types); and T_cell_CD8 (R = -0.34, P < 0.001) and DC (R = -0.25, P < 0.001) in TIMER (6 cell types). (**Figure 5A**).

Pearson correlation analysis revealed a significant association between the levels of DDR1 and those of 63 immunoregulatory genes (P < 0.05), with the strongest correlations observed for CD276 (R = 0.48, P < 0.001), VTCN1 (R = 0.41, P < 0.001), and SLAMF7 (R = -0.37, P < 0.001) (**Figure 5B**).

### 3.5 Drug sensitivity analysis based on DDR1

**Figure 6:**
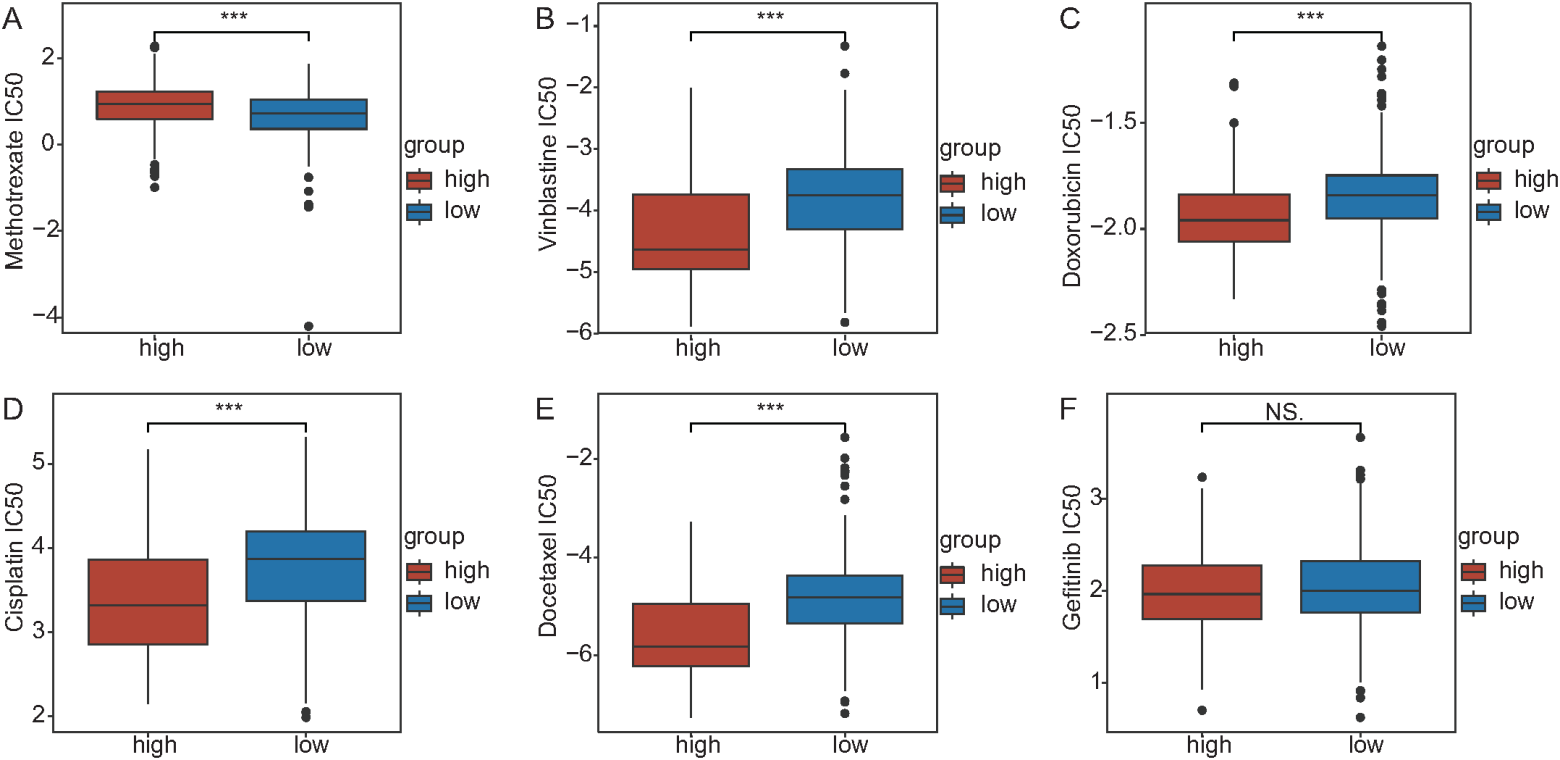
Drug sensitivity analysis. Differences in the IC50 values for methotrexate **(A)**, vinblastine **(B)**, doxorubicin **(C)**, cisplatin **(D)**, docetaxel **(E)**, and gefitinib **(F)** between the high and low DDR1 expression groups.

**Figure 7:**
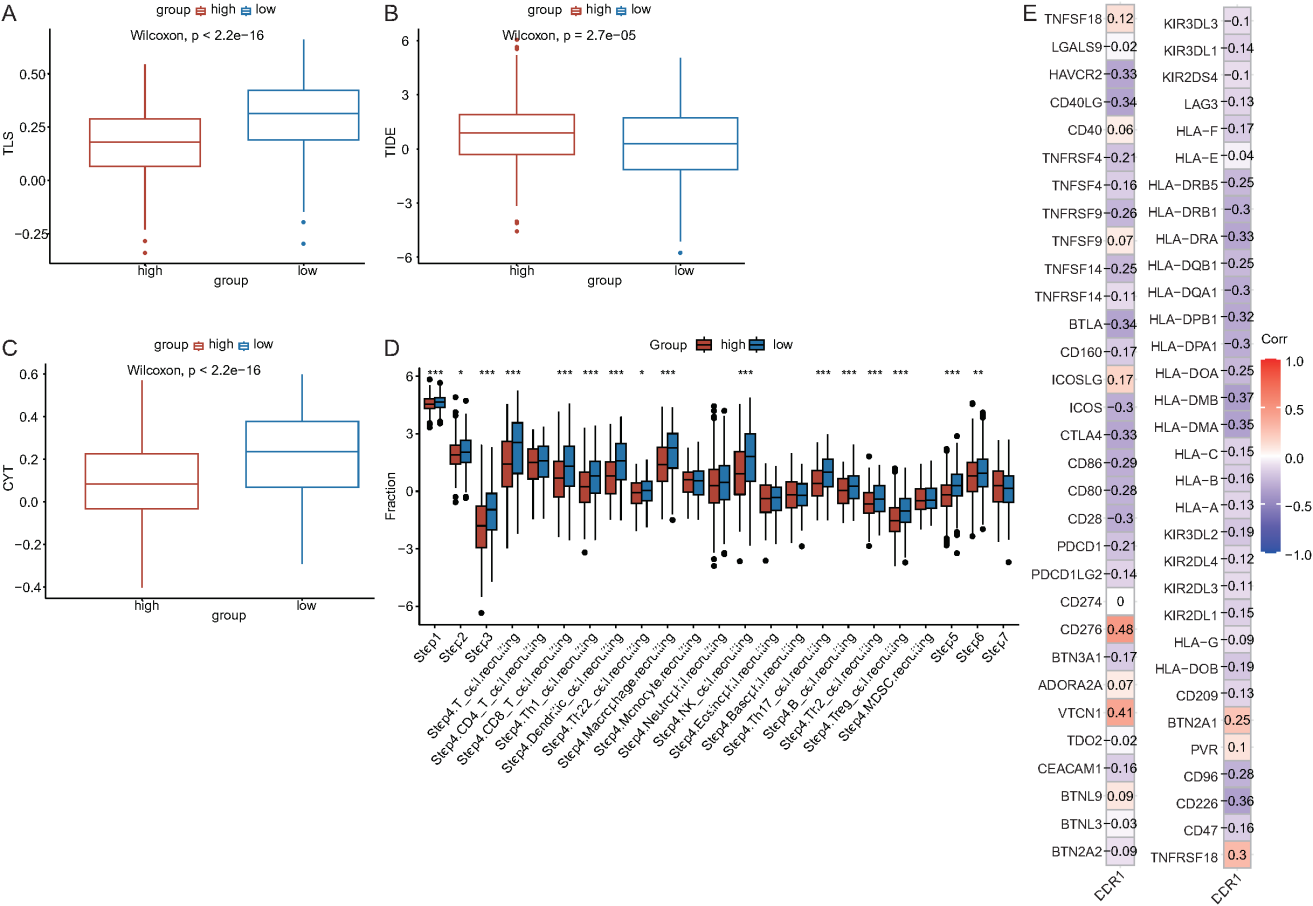
Correlation analysis of immune therapy predictors. Distribution of TLS **(A)**, TIDE **(B)** and CYT **(C)** scores between the high and low DDR1 expression groups. **(D)** Differences in tumor immune cycle steps between the high and low DDR1 expression groups. **(E)** Correlations between immune checkpoint genes and DDR1 expression.

The IC50 values of chemotherapy drugs (methotrexate, vinblastine, doxorubicin, cisplatin, docetaxel, and gefitinib) were calculated in NSCLC patients. Methotrexate had a greater IC50 in the high DDR1 expression group (P < 0.05) (**Figure 6A**), whereas vinblastine, doxorubicin, cisplatin, and docetaxel had lower IC50 values in the high DDR1 expression group (P < 0.05) (**Figures 7B–E**). No significant difference in the IC50 was observed for gefitinib between the two groups (P > 0.05) (**Figure 6F**).

### 3.6 DDR1-based immunotherapy response prediction

To investigate the role of cell death index features in the immune therapy response, we examined the relationships between DDR1 and immune therapy predictors (TLS, TIDE, and CYT). TLS was higher in the low DDR1 expression group (p < 0.05) (**Figure 7A**), whereas TIDE was higher in the high DDR1 expression group (P < 0.05) (**Figure 7B**). CYT was higher in the low DDR1 expression group (P < 0.05) (**Figure 7C**). Tumor immune cycle analysis revealed significant differences in 16 steps between the high and low DDR1 expression groups of NSCLC patients (P < 0.05) (**Figure 7D**), with higher proliferation levels in the high-risk group. Pearson correlation analysis revealed the strongest positive correlation between CD276 and DDR1 levels (R = 0.48, P < 0.05) and the strongest negative correlation between HLA-DMB and DDR1 levels (R = -0.37, P < 0.05) (**Figure 7E**).

### 3.7 Single-cell heterogeneity analysis

**Figure 8:**
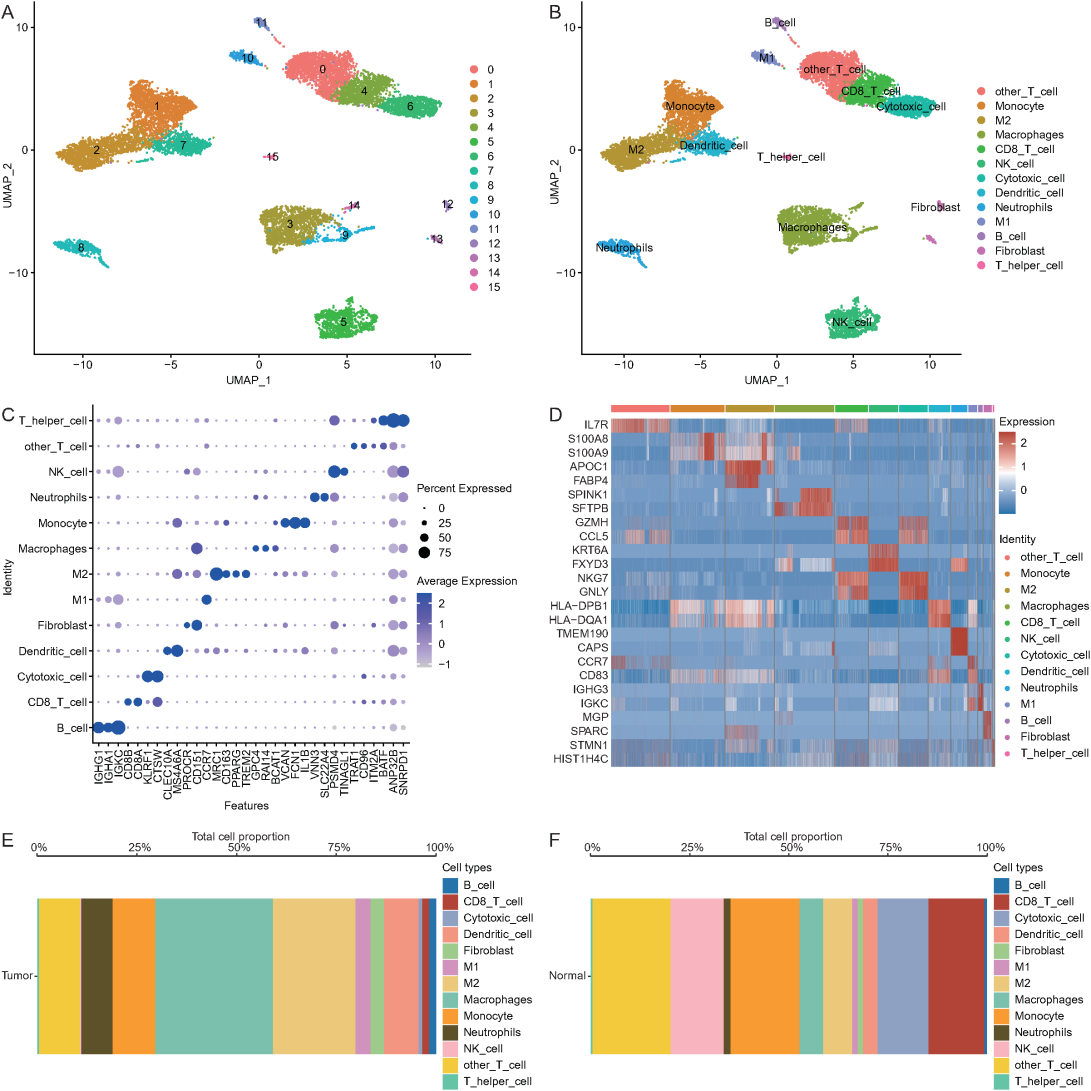
Single-cell heterogeneity. **(A)** Clustering results for single-cell data. **(B)** Cell type annotation results. **(C)** Bubble plot of marker gene expression across different cell types. **(D)** Heatmap of differentially expressed genes in the single-cell transcriptome (red indicates upregulated expression, blue indicates downregulated expression). **(E)** Proportions of cell types in tumor samples. **(F)** Proportions of cell types in normal samples.

UMAP clustering of the NSCLC single-cell dataset revealed 16 clusters (**Figure 8A**), and manual annotation revealed 13 cell types (B cells, CD8 T cells, cytotoxic cells, dendritic cells, fibroblasts, M1 cells, M2 cells, macrophages, monocytes, neutrophils, NK cells, other T cells, and T helper cells) (**Figure 8B**). Marker gene expression across cell types was visualized in a bubble plot (**Figure 8C**). Differential expression analysis and a heatmap (**Figure 8D**) highlighted the top two genes with the most significant expression differences. The proportions of each cell type are shown in bar charts (**Figures 8E, F**), revealing that macrophages predominated in the tumor samples, whereas other T cells were most abundant in the normal samples.

### 3.8 Pseudotime analysis of single-cell data

**Figure 9:**
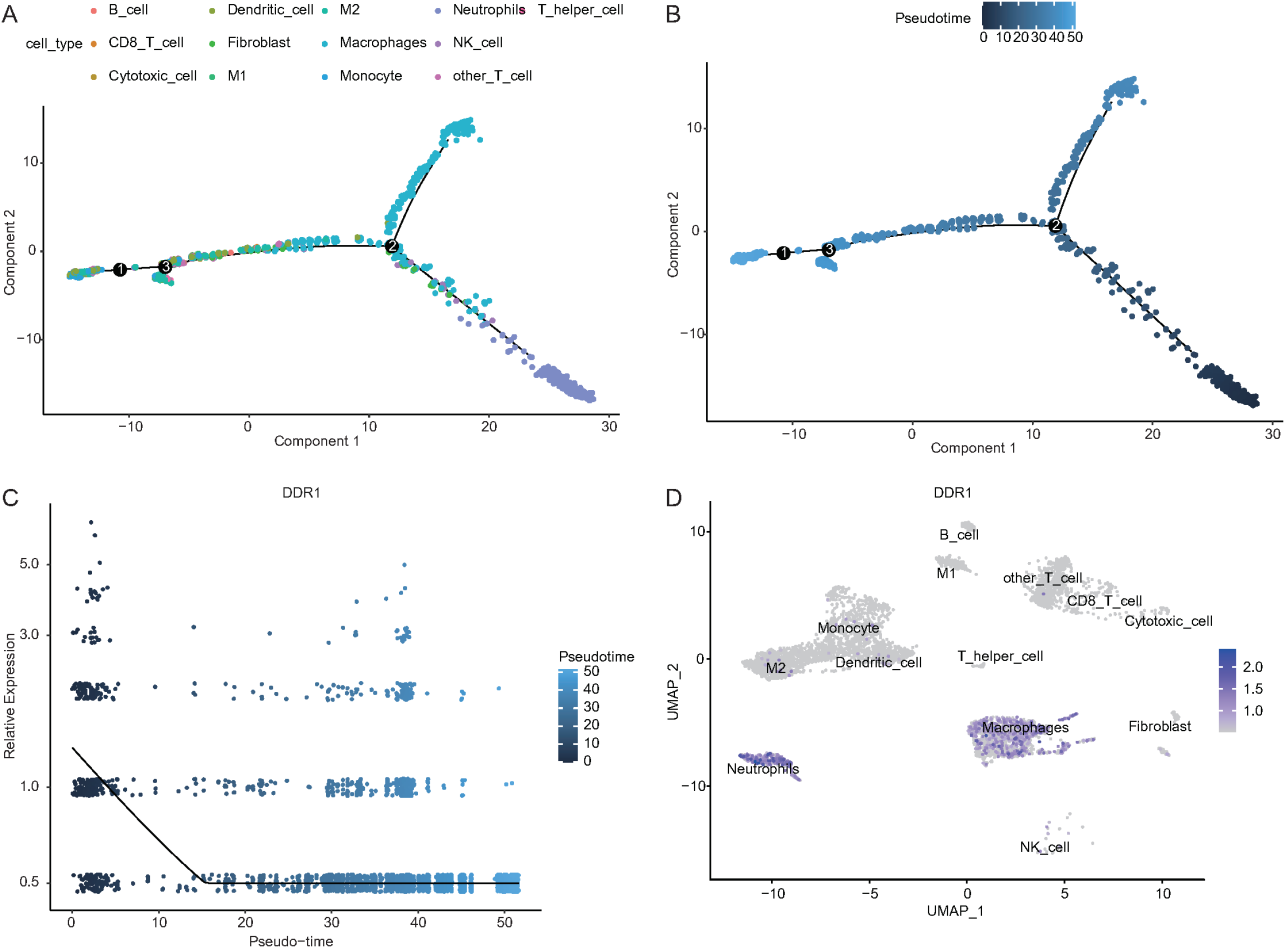
Pseudotime analysis. **(A)** Differentiation trajectory of 13 cell subpopulations in tumor samples (colors represent different cell subtypes). **(B)** Developmental timeline of 13 cell subpopulations (color intensity from dark to light represents pseudotime). **(C)** Timeline showing changes in DDR1 expression during the developmental trajectory. **(D)** UMAP plot showing DDR1 expression across different cell types.

Pseudotime analysis was conducted to explore the developmental trajectories of 13 cell types in tumor samples, as shown in the differentiation trajectory plot (**Figure 9A**) and timeline plot (**Figure 9B**). DDR1 expression decreased along the developmental trajectory (**Figure 9C**). A UMAP plot further revealed differential DDR1 expression among cell types, with notably higher expression in macrophages (wilcox test: avg_log2FC = 1.87, P_val_adj < 0.001) and neutrophils (wilcox test: avg_log2FC = 2.74, P_val_adj < 0.001) (**Figure 9D**).

### 3.9 CellChat analysis of cell communication

**Figure 10:**
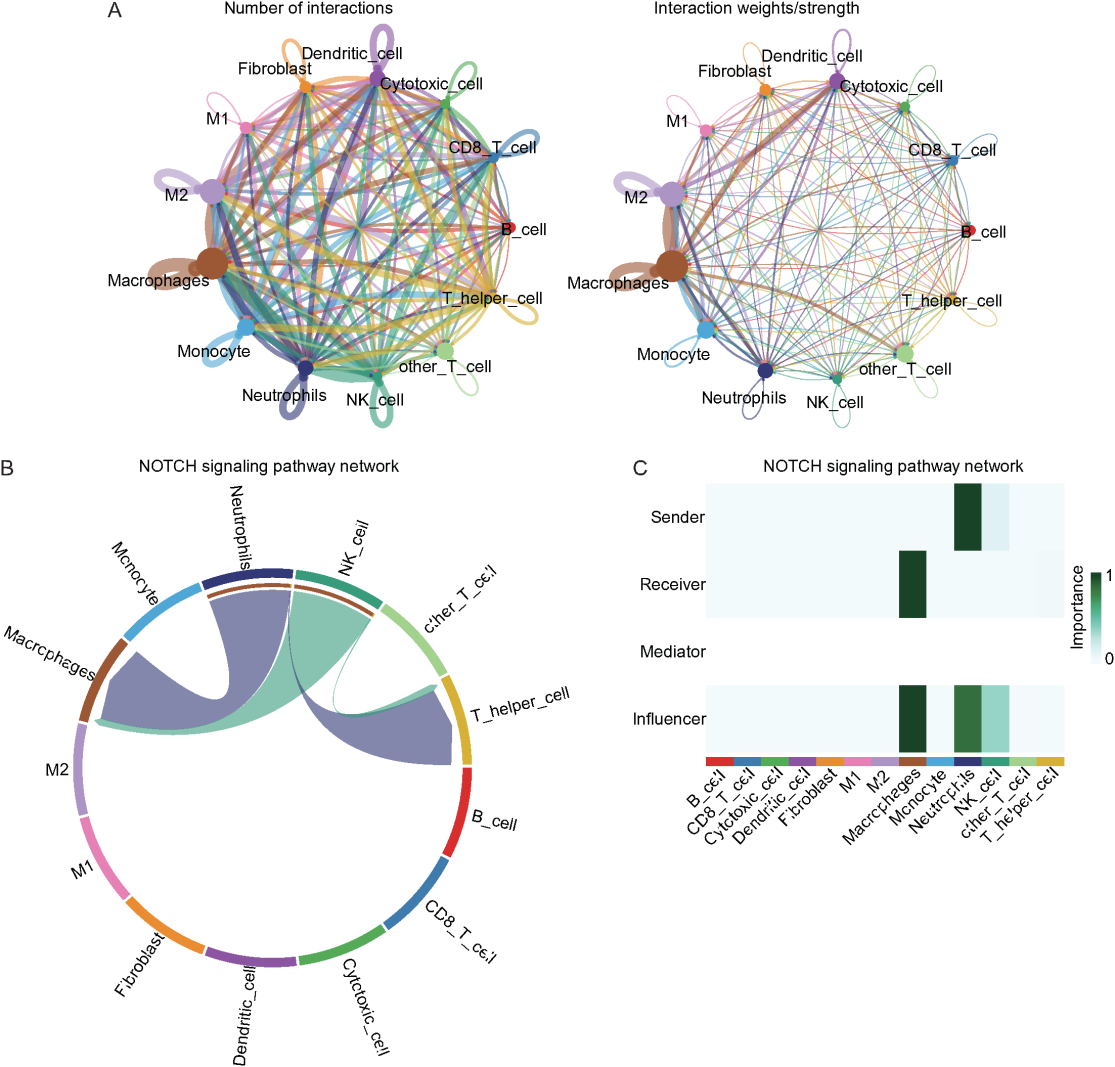
Cell communication analysis between macrophages, neutrophils, and other cell types. **(A)** Interactions between different cell types in tumor samples. **(B)** Predicted NOTCH signaling network. The size of the circles is proportional to the number of cells in each group, and the edge width indicates the communication probability. **(C)** Heatmap showing the relative importance of each cell group on the basis of four network centrality metrics calculated from the NOTCH signaling network.

**Figure 11:**
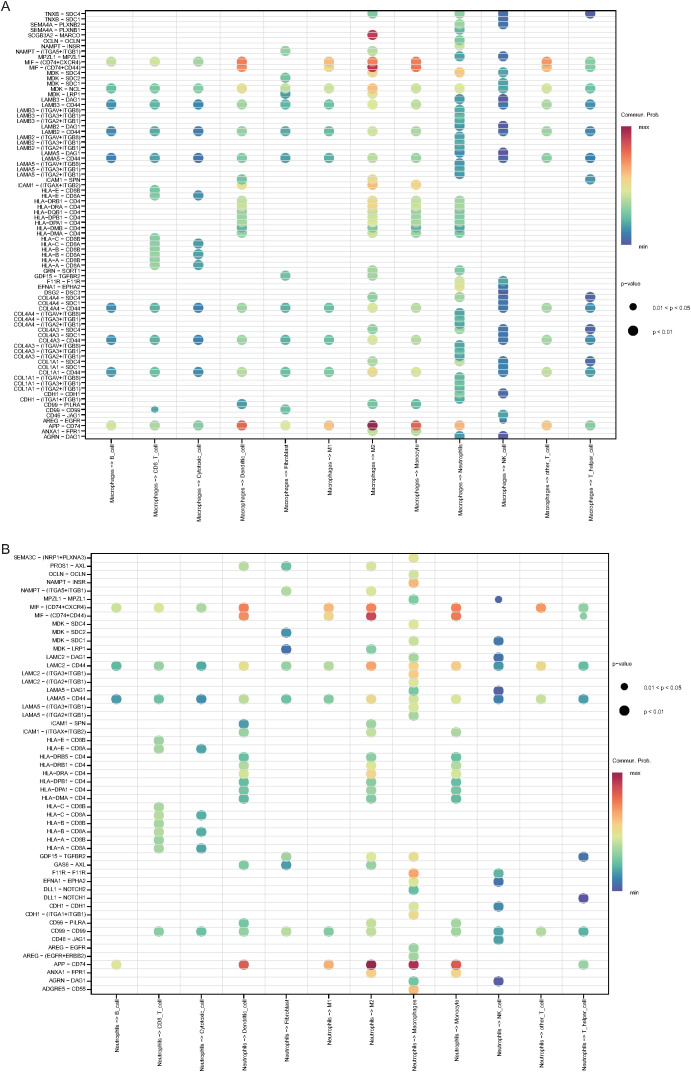
Receptor–ligand interactions between macrophages, neutrophils, and other cell types. Bubble plot of receptor–ligand interactions between macrophages **(A)**, neutrophils **(B)** and other cell types (color intensity indicates interaction strength, and bubble size reflects significance).

To better understand the interactions between macrophages, neutrophils, and other cell types, CellChat analysis was conducted on tumor samples. The results revealed a close connection between these cell types (**Figure 10A**). The NOTCH signaling pathway was identified as a key pathway linking macrophages and neutrophils (**Figure 10B**). Network centrality analysis revealed that neutrophils likely act as senders, whereas macrophages function as receivers in the NOTCH pathway (**Figure 10C**). Further analysis of the receptor–ligand interactions, visualized in bubble plots, revealed that the ANXA1–FPR1 pair represented a strong interaction between macrophages and M2 macrophages (**Figure 11A**), and the APP–CDD74 pair represented a strong interaction between neutrophils and M2 macrophages (**Figure 11B**).

### 3.10 Investigation of potential biological mechanisms enriched in the high and low DDR1 expression groups

**Figure 12:**
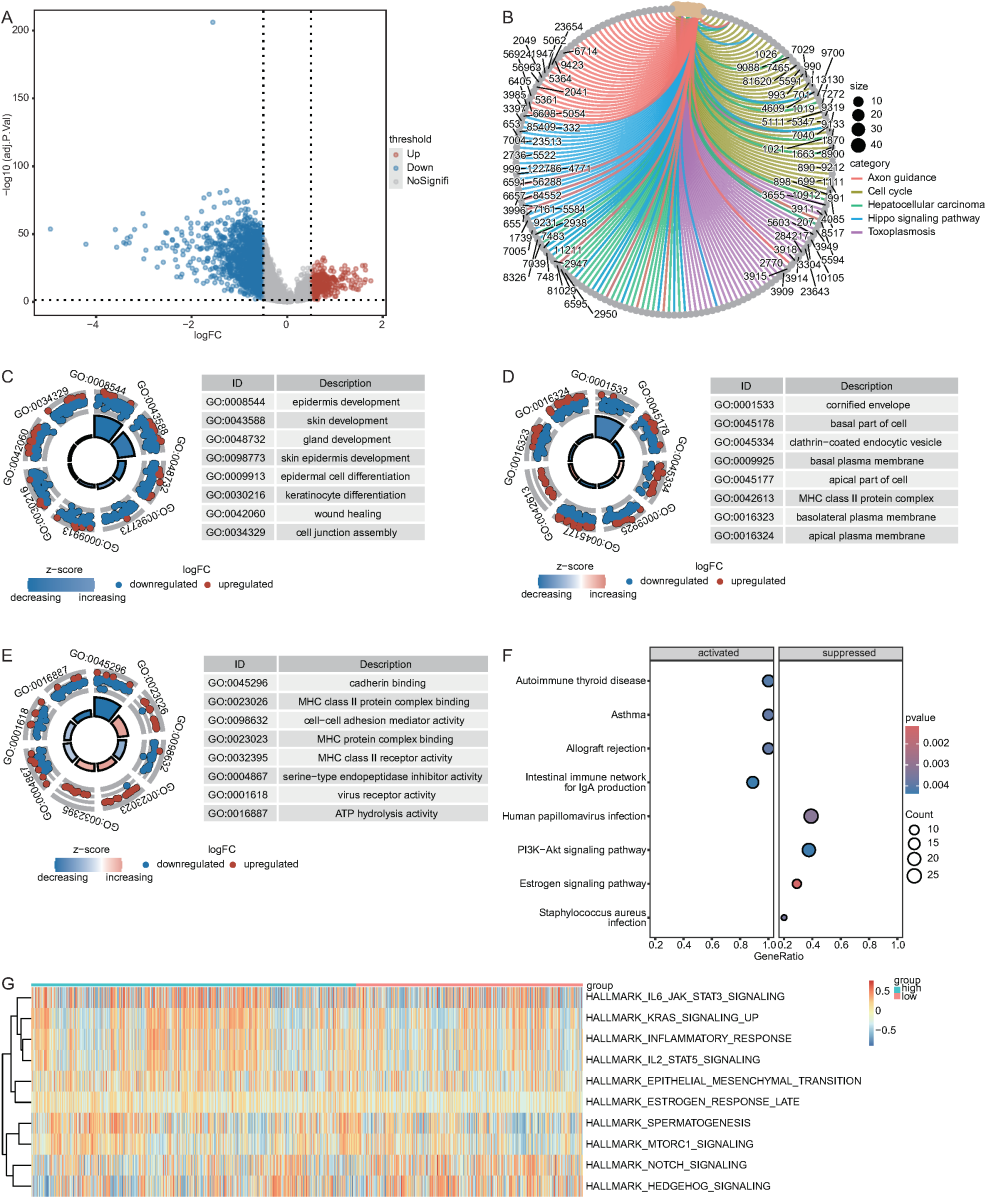
Enrichment analysis between the high and low DDR1 expression groups. **(A)** Volcano plot of the differential expression analysis results (red: upregulated; blue: downregulated). **(B–G)** Enrichment analysis results for KEGG analysis, BP analysis, CC analysis, MF analysis, GSVA, and GSEA.

To compare gene expression between the high and low DDR1 expression groups, we identified 382 upregulated and 1,749 downregulated DEGs in NSCLC tumor samples from the TCGA database (**Figure 12A**) (**Supplementary Table 2**). The results of the GO and KEGG enrichment analyses are shown in **Supplementary Table 3** and **Supplementary Table 4**. The DEGs were associated with pathways such as the cell cycle, toxoplasmosis, and hepatocellular carcinoma (**Figure 12B**); biological processes such as epidermis development, skin development and gland development (**Figure 12C**); cellular components such as the cornified envelope, basal part of the cell and clathrin-coated endocytic vesicle (**Figure 12D**); and molecular functions such as cadherin binding, MHC class II protein complex binding and cell–cell adhesion mediator activity (**Figure 12E**).

GSEA enrichment analysis was performed on the basis of the log2FoldChange values of the DEGs from the differential analysis. The results are shown in **Supplementary Table 5**. **Figure 12F** displays the top four most significant pathways in terms of both activation and inhibition, including autoimmune thyroid disease, asthma, allograft rejection, the intestinal immune network for IgA production, the estrogen signaling pathway, human papillomavirus infection, the PI3K-Akt signaling pathway, and Staphylococcus aureus infection.

GSVA was performed on common pathways for the high and low DDR1 expression groups, followed by differential analysis of pathway scores using the limma package. The results are shown in the heatmap. All GSVA results are provided in **Supplementary Table 6**. GSVA revealed significant differences (p < 0.05) in the enrichment of gene sets such as HALLMARK_SPERMATOGENESIS, HALLMARK_NOTCH_SIGNALING, and HALLMARK_HEDGEHOG_SIGNALING between the high and low DDR1 expression groups (**Figure 12G**).

### 3.11 Construction of the prognostic model for NSCLC

**Figure 13:**
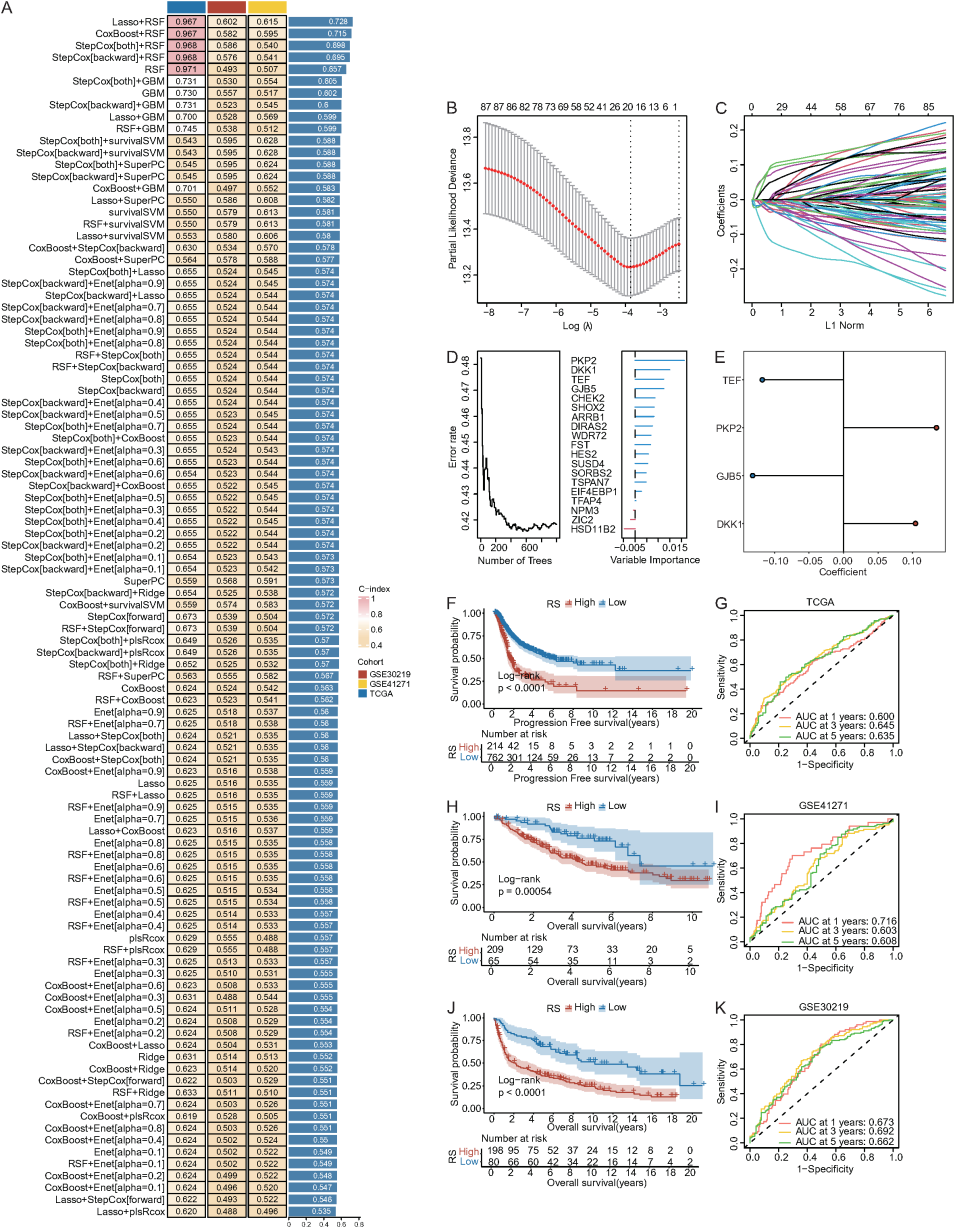
Construction of the prognostic model for NSCLC. **(A)** C-index of 101 algorithm combinations in 4 cohorts. **(B, C)** LASSO analysis. **(D)** RSF analysis. **(E)** Risk regression coefficients for 4 genes. **(F)** Survival curves of high/low RiskScore groups in TCGA; **(G)** ROC analysis for 1-, 3-, and 5-year survival in the TCGA cohort. **(H)** Survival curves in the GSE41271 cohort. **(I)** ROC analysis in the GSE41271 cohort. **(J)** Survival curves in the GSE30219 cohort. **(K)** ROC analysis in the GSE30219 cohort.

On the basis of the DEGs identified according to the expression levels of DDR1 mentioned above, univariate Cox analysis was performed on the DEGs in both the training and validation datasets, identifying 88 genes with consistent hazard ratios (P < 0.05) across two or more datasets. In the TCGA-NSCLC cohort, 101 algorithm combinations were used to construct a predictive model via 10-fold cross-validation. The robustness of the model was evaluated in multiple validation cohorts, and the LASSO + RSF model, with the highest average C-index (0.728), was selected (**Figure 13A**). LASSO analysis revealed 19 genes (ARRB1, CHEK2, DIRAS2, DKK1, EIF4EBP1, FST, GJB5, HES2, HSD11B2, NPM3, PKP2, SHOX2, SORBS2, SUSD4, TEF, TFAP4, TSPAN7, WDR72, and ZIC2) (**Figures 13B, C**), and RSF analysis revealed the importance of these genes (**Figure 13D**). Four genes with importance scores >0.01 were selected as prognostic genes (**Figure 13E**). Risk regression coefficients were calculated using the proportional hazards model, and the RiskScore formula was constructed accordingly. The formula was as follows:

RiskScore = (0.135 * PKP2 exp.) +(0.104 * DKK1 exp.) +(-0.118 * TEF exp.) +(-0.132 * GJB5 exp.).

Risk scores ranged from 0.2 to 2.1, with the median value of 1.0 used as the cutoff to classify patients into high- and low-risk groups. After the RiskScore for each NSCLC patient was calculated, patients were grouped according to the cutoff from the survminer package (**Supplementary Table 7**). Survival analysis revealed that the low-RiskScore group had significantly better survival in the TCGA cohort (P < 0.05) (**Figure 13F**), as well as in the GSE41217 (P < 0.05) (**Figure 13H**) and GSE30219 cohorts (P < 0.05) (**Figure 13J**). The performance of the model was evaluated, revealing good results in the TCGA cohort, with 1/3/5-year AUCs of 0.600/0.645/0.663 (**Figure 13G**), and similar results in the GSE41217 (0.716/0.603/0.608) (**Figure 13I**) and GSE30219 (0.673/0.692/0.662) cohorts (**Figure 13K**). These findings confirm the stable performance of our model across multiple cohorts.

### 3.12 Comparison of the NSCLC prognostic model with other signatures

To comprehensively compare the performance differences between our prognostic model and other signatures, we systematically reviewed published prognostic signatures or models. A total of 38 signatures, including those for LUAD, LUSC, and NSCLC, were included in this study. Univariate Cox regression analysis revealed that our prognostic model can serve as a risk factor for NSCLC prognosis in the TCGA, GSE30219, and GSE41217 cohorts (**Supplementary Figure 2A**). We then calculated the C-index values for the 38 signatures across the three cohorts. The results revealed that in the TCGA cohort (**Supplementary Figure 2B**), GSE30219 cohort (**Supplementary Figure 2C**), and GSE41217 cohort (**Supplementary Figure 2D**), our model consistently ranked among the top signatures, outperforming the majority of previously published signatures.

### 3.13 Knockdown of DDR1 *in vitro* inhibits the proliferation of lung cancer cells

**Figure 14:**
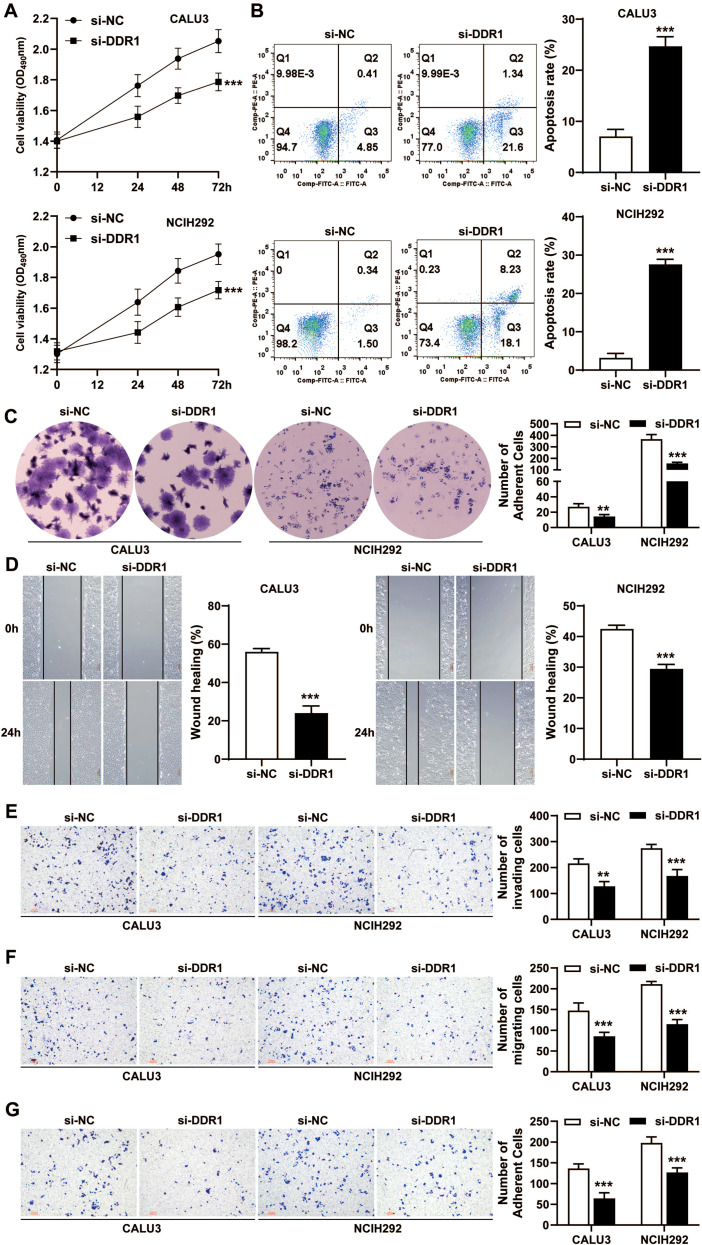
Effect of DDR1 knockdown on the proliferation, invasion, migration, and adhesion of CALU3 and NCI-H292 cells. A cell viability assay **(A)**, apoptosis analysis by flow cytometry **(B)**, and a colony formation assay **(C)** demonstrated changes in cell proliferation following DDR1-siRNA transfection. Scratch assays **(D)** revealed that DDR1 knockdown significantly reduced the migration ability of CALU3 and NCI-H292 cells. Transwell assays **(E, F)** revealed a marked decrease in the number of cells that crossed the Transwell membrane, indicating a significant reduction in migration and invasion capacity. Furthermore, an adhesion assay **(G)** demonstrated that DDR1 knockdown led to a substantial decrease in the adhesion of CALU3 and NCI-H292 cells to the extracellular matrix. ***P<0.001, **P<0.01, *P<0.05. NC, negative control; si, small interfering; OD, optical density.

To investigate the role of DDR1 in lung cancer cell proliferation, invasion, migration, and adhesion, we performed siRNA-mediated knockdown of DDR1 in NSCLC cell lines. RT–qPCR, western blotting, and immunofluorescence confirmed DDR1 expression in HARA, CALU3, and NCI-H292 cells (**Supplementary Figures 3A–C**). CALU3 and NCIH292 cells were selected for subsequent experiments. The knockdown efficiency of DDR1-siRNA was validated by RT–qPCR and western blotting, which revealed significant suppression of DDR1 expression in CALU3 and NCI-H292 cells (P<0.05) (**Supplementary Figures 3D–G**). MTT, apoptosis, and colony formation assays revealed a significant reduction in the proliferative capacity of CALU3 and NCI-H292 cells following DDR1-siRNA transfection (**Figures 14A–C**). DDR1 depletion significantly impaired cell migration, invasion, and adhesion, as demonstrated by the results of the scratch, Transwell, and adhesion assays (**Figures 14D–G)**.

### 3.14 Pathological examination of clinical samples

**Figure 15:**
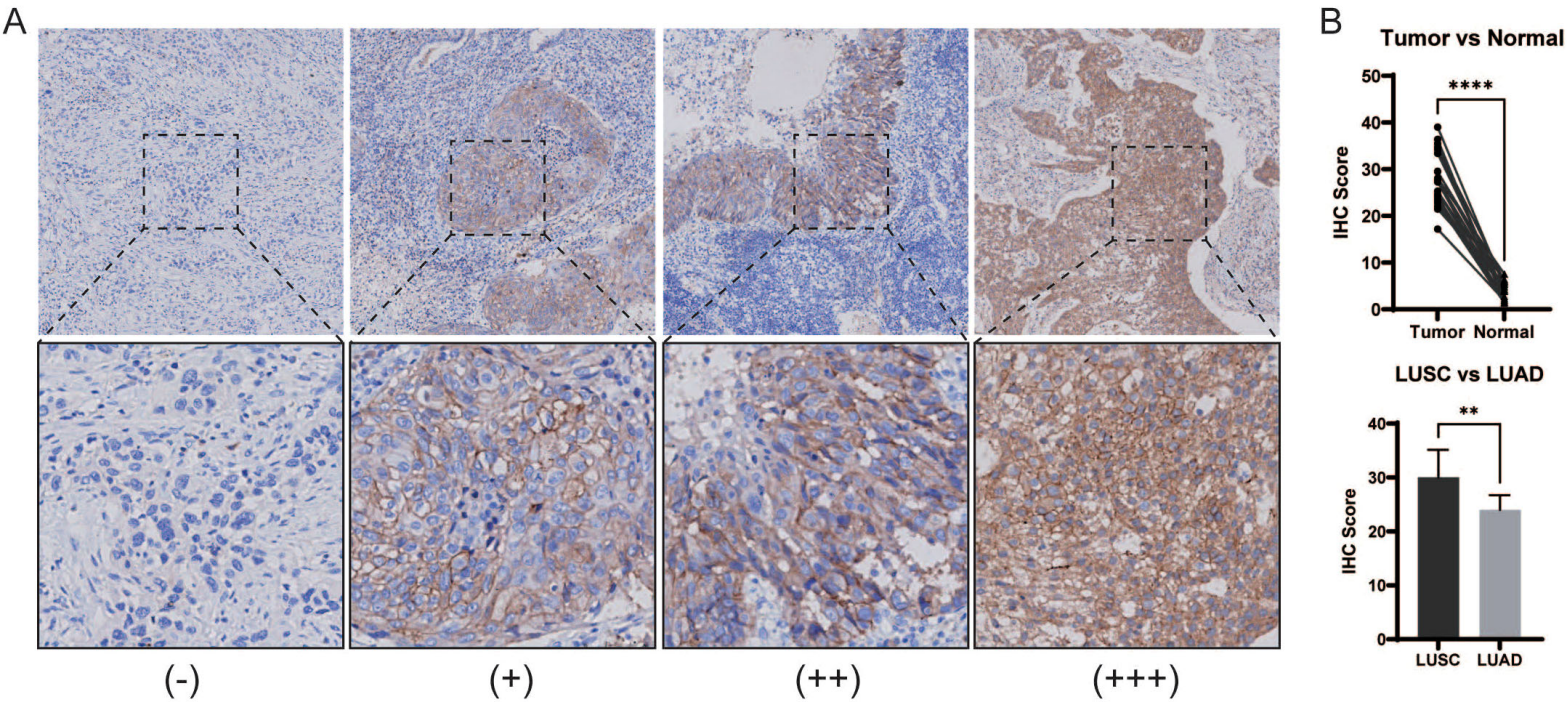
DDR1 expression in NSCLC tumor tissue. **(A)** Representative IHC images of DDR1 expression in NSCLC tissues. -: negative staining, +: mild positive staining, ++: moderate positive staining, +++: strong positive staining. Magnification, up 20×; down 80×. **(B)** Comparison of the IHC scores of DDR1 in NSCLC tumor tissues and paired adjacent normal tissues**** P < 0.0001. **(C)** Comparison between LUSC and LUAD. ** P < 0.01.

A total of 34 NSCLC samples were included in this study, 19 (55.88%, 19/34, mean ± SD age, 63.84 ± 9.32 years; 2 females, 17 males) of which were positive. Among them, 5 of 17 LUAD cases (29.41%) and 14 of 16 LUSC cases (87.50%) were positive for DDR1. The IHC score of DDR1 in positive samples was significantly greater than that of adjacent normal tissues (27.84 ± 5.815 *vs*. 3.734 ± 1.895, P < 0.0001). Moreover, the IHC score DDR1 in in LUSC was significantly greater than that in LUAD (30.03 ± 5.102 *vs*. 24.02 ± 2.689, *P = 0.0062) (**Figure 15**).

## 4 Discussion

This study systematically explores DDR1’s role in NSCLC progression and prognosis via bioinformatics, immunohistochemistry of clinical samples, and *in vitro* experiments. Elevated DDR1 expression was linked to advanced tumor stage (T3), older age (>65), female sex, and shorter progression-free survival, highlighting its prognostic relevance. Multivariate analysis identified DDR1 and pathological stage as independent prognostic factors, supporting the construction of a predictive nomogram. Our findings align with Sun Ho Yang et al (62), who reported DDR1 overexpression in 61% of NSCLC cases, especially invasive adenocarcinoma. In comparison, our study observed DDR1 expression in 55.88% of cases—likely due to sample size differences. Additionally, univariate analysis showed high DDR1 expression correlated with poorer overall survival. Previous studies have shown that collagen I-activated DDR1 promotes NSCLC cell migration and invasion by upregulating MMP-2, N-cadherin, and vimentin (63).

Our analysis revealed that DDR1 expression in NSCLC is significantly negatively correlated with DNA methylation levels (R = -0.43, P < 0.05), suggesting that epigenetic regulation is a potential driver of DDR1 dysregulation in tumors. Global DNA hypomethylation and promoter hypermethylation-induced gene inactivation are well-established epigenetic hallmarks of cancer cells (64, 65). Low-methylation epigenotypes are associated with a poorer prognosis for LUSC (66). An inverse relationship between DDR1 promoter methylation and DDR1 expression was observed at the five CpG sites previously analyzed in NSCLC, with hypomethylation identified as an independent prognostic factor for disease-free survival (67). Studies on DDR1 methylation remain limited, and further research is needed to elucidate its underlying mechanisms.

Notably, although DDR1 mutations were detected in the NSCLC cohorts, no significant differences in survival or expression were detected between the mutant and wild-type groups (P > 0.05), and the difference in TMB between the DDR1 expression groups was not significant (P > 0.05). These findings suggest that DDR1 mutation or expression may not be the primary drivers of its oncogenic activity in NSCLC and that DDR1-driven tumor progression may rely more on microenvironmental interactions (e.g., ECM remodeling) rather than intrinsic genomic instability, with DDR1 overexpression promoting collagen alignment and creating a barrier to immune infiltration independent of mutational burden (10). Our *in vitro* cellular experiments further confirmed that DDR1 knockdown inhibits the proliferation and migration of NSCLC cells.

Comparative somatic mutation profiling revealed distinct mutation patterns between the high and low DDR1 expression groups. High DDR1 expression was associated with amplifications on chromosomes 3, 8, and 11, which harbor key oncogenes such as PIK3CA on chromosome 3 and MYC on chromosome 8 (68). Notably, PIK3CA genomic gain, as detected by FISH, has been reported in 43% of lung cancers, with a preference for squamous cell carcinoma (69, 70). Additionally, deletions on chromosomes 2 and 9 impact key tumor suppressors, including the well-known CDKN2A on chromosome 9 (71). The activation or amplification of these oncogenes and the deletion of tumor suppressors play crucial roles in lung cancer pathogenesis. Conversely, low DDR1 expression cohorts presented amplifications of chromosomes 1 and 14, potentially linked to alternative protumorigenic pathways such as MDM4 (chr1) or AKT1 (chr14) activation (72, 73).

Further multialgorithm analyses revealed that elevated DDR1 expression is significantly correlated with immune cell infiltration in NSCLC, which may play a crucial role in tumor promotion and immune escape. In addition to the aforementioned role of DDR1 in influencing the TME by promoting collagen realignment and restricting immune cell infiltration, the significant upregulation of immune checkpoint genes in the high DDR1 expression group may enhance immune evasion by inhibiting T-cell function. Notably, the association of DDR1 with 63 immunoregulatory genes underscores its central role in tumor immune regulation. For example, DDR1 modulates the MMP and chemokine secretion to recruit macrophages, shaping an immunosuppressive TME, a mechanism validated in hepatic metastasis models where DDR1 silencing reduces MMP2/9 and prometastatic factors in HSCs, thereby inhibiting tumor growth and immune escape (74).

Drug sensitivity analysis revealed that the high DDR1 expression group presented increased IC50 values for methotrexate but increased sensitivity to vinblastine, doxorubicin, cisplatin and docetaxel. This may be attributed to DDR1-driven collagen barriers, which promote drug efflux and contribute to matrix-mediated drug resistance (75). DDR1 inhibitors can overcome ECM-mediated drug resistance by disrupting DDR1/PYK2/FAK signaling, remodeling the tumor microenvironment, and enhancing the efficacy of conventional chemotherapeutic agents (76, 77). However, these findings are based on preclinical studies and warrant further investigation in clinical settings. With respect to immunotherapy prediction, elevated TIDE scores in the high DDR1 expression group suggest an immune evasion phenotype, whereas reduced CYT scores reflect impaired cytotoxic T-cell activity. These findings align with preclinical studies of DDR1 inhibitory antibodies: ECD-neutralizing antibodies disrupt collagen fiber alignment, mitigate immune exclusion and inhibit tumor growth in immunocompetent hosts (10). The above analyses highlight the immunotherapeutic potential of targeting DDR1 and identifying promising DDR1-directed therapeutic agents; however, rigorous preclinical and clinical validation of these findings is imperative.

UMAP-based clustering of the single-cell heterogeneity data revealed 16 clusters corresponding to 13 major cell types, highlighting the complexity of the NSCLC TME. Notably, macrophages were found to be the predominant cell type in tumor tissues, whereas T cells were more enriched in normal tissues, which is consistent with previous reports suggesting macrophage enrichment and T-cell abnormalities in NSCLC tumors (78, 79). Pseudotime analysis revealed decreasing DDR1 expression along progression trajectories, with higher levels in macrophages/neutrophils. This dynamic pattern positions DDR1 as a regulator of NSCLC microenvironment remodeling, suggesting that coordinated changes in immune cell composition (notably immunosuppressive shifts) are linked to both immune evasion and fibrosis-like ECM reorganization (80). CellChat analysis revealed macrophages and neutrophils as central NOTCH signaling mediators, with neutrophils functioning as senders and macrophages as receivers, which aligns with known mechanisms of macrophage polarization and myeloid-derived suppressor cell (MDSC) recruitment (81). Key ligand–receptor pairs (ANXA1–FPR1 and APP–CD74) that drive these interactions regulate the phenotypes of tumor-associated macrophages (TAMs), which are functionally linked to the TME (82, 83). Enrichment analysis of the DDR1-high and DDR1-low groups revealed enrichment of the cell cycle and PI3K–Akt signaling pathways, both of which are well-established drivers of tumor proliferation, survival, and therapeutic resistance in NSCLC (84, 85). GSVA/GSEA revealed that DDR1-high-expressing tumors are associated with developmental oncogenic programs (NOTCH/Hedgehog), pathways mechanistically linked to cell fate regulation (proliferation/differentiation/apoptosis) and core tumorigenic processes (invasion/migration) (86, 87). Together, these data suggest that DDR1 is a regulator of the ECM and cellular adhesion and a key factor in the TME.

In this study, we developed a robust prognostic model for NSCLC by integrating the LASSO and random survival forest algorithms and identified four key genes (PKP2, DKK1, TEF, and GJB5). The combined expression of these genes effectively stratified patients into high- and low-risk groups with significantly different survival outcomes. The model demonstrated strong and consistent predictive performance across the TCGA, GSE41217, and GSE30219 cohorts, underscoring its generalizability and clinical potential. Both PKP2 and DKK1 promote the development and progression of NSCLC (88, 89). TEF and GJB5 appeared to function as protective factors in our model. Emerging evidence indicates that TEF expression is lower in NSCLC tissues than in normal tissues and that TEF expression is positively correlated with better clinical survival rates (90). In bladder cancer, upregulation of TEF expression significantly retarded bladder cancer cell growth by inhibiting the G1/S transition via regulating AKT/FOXOs signaling (91). GJB5, a gap junction protein, is traditionally viewed as a tumor suppressor, and its loss of connexin-mediated communication is linked to cancer progression (92–94). Overexpression of GJB5 in NSCLC cells reduced cell proliferation, induced a delay in the G1 phase, inhibited anchorage-independent growth and suppressed cell migration and invasion (95). However, the role of TEF and GJB5 in NSCLC remains underexplored and warrants further investigation. Furthermore, our comparison with 38 published prognostic signatures demonstrated that our model ranked first in the TCGA cohort, eighth in the GSE30219 dataset, and thirteenth in the GSE41271 dataset, indicating robust and consistent predictive performance across multiple independent cohorts. This finding supports prior findings that combining robust feature selection with machine learning enhances model performance and reproducibility in high-dimensional omics data (18).

Notably, our study has several limitations. First, our findings are derived from bioinformatics analyses of publicly available datasets and require validation in real-world patient cohorts. Variations in datasets and analytical approaches may lead to inconsistent results, even when the same pathological condition is investigated (96). Second, the prognostic significance of DDR1 in the context of immunotherapy warrants prospective evaluation in larger patient populations. Finally, the number of pathological samples analyzed in this study is relatively limited, underscoring the need for further investigations with expanded sample sizes to better elucidate the role of DDR1 in NSCLC.

## 5 Conclusion

In conclusion, our research indicates that DDR1 is upregulated in NSCLC and closely associated with a poor prognosis, serving as one of the important driving factors for the development of NSCLC. *In vitro* validation and pathological analysis revealed that DDR1 has a significant effect on the biological function of NSCLC cancer cells, promoting tumor cell proliferation, migration and invasion. Notably, we further revealed potential biological mechanisms and identified potential therapeutic drugs that may function in individuals with high DDR1 expression. In addition, through systematic integration of DEGs selected on the basis of DDR1 expression profiles, we developed a robust machine learning-driven prognostic model that demonstrated predictive performance superior to that of traditional models. These results highlight the potential of DDR1 as a therapeutic strategy for treating NSCLC.

## Data Availability

The original contributions presented in the study are included in the article/**Supplementary Material**. Further inquiries can be directed to the corresponding authors.
