# Supplementary material for: DDR1 as a key prognostic biomarker in non-small cell lung cancer: identification, validation, and potential therapeutic implications
Source: Front Immunol. 2025 Nov 28;16:1690829. doi: 10.3389/fimmu.2025.1690829 (PMC12698539; doi:10.3389/fimmu.2025.1690829)
Supplement: Supplementary file 8 [file DataSheet1.pdf]

Supplementary Figures:

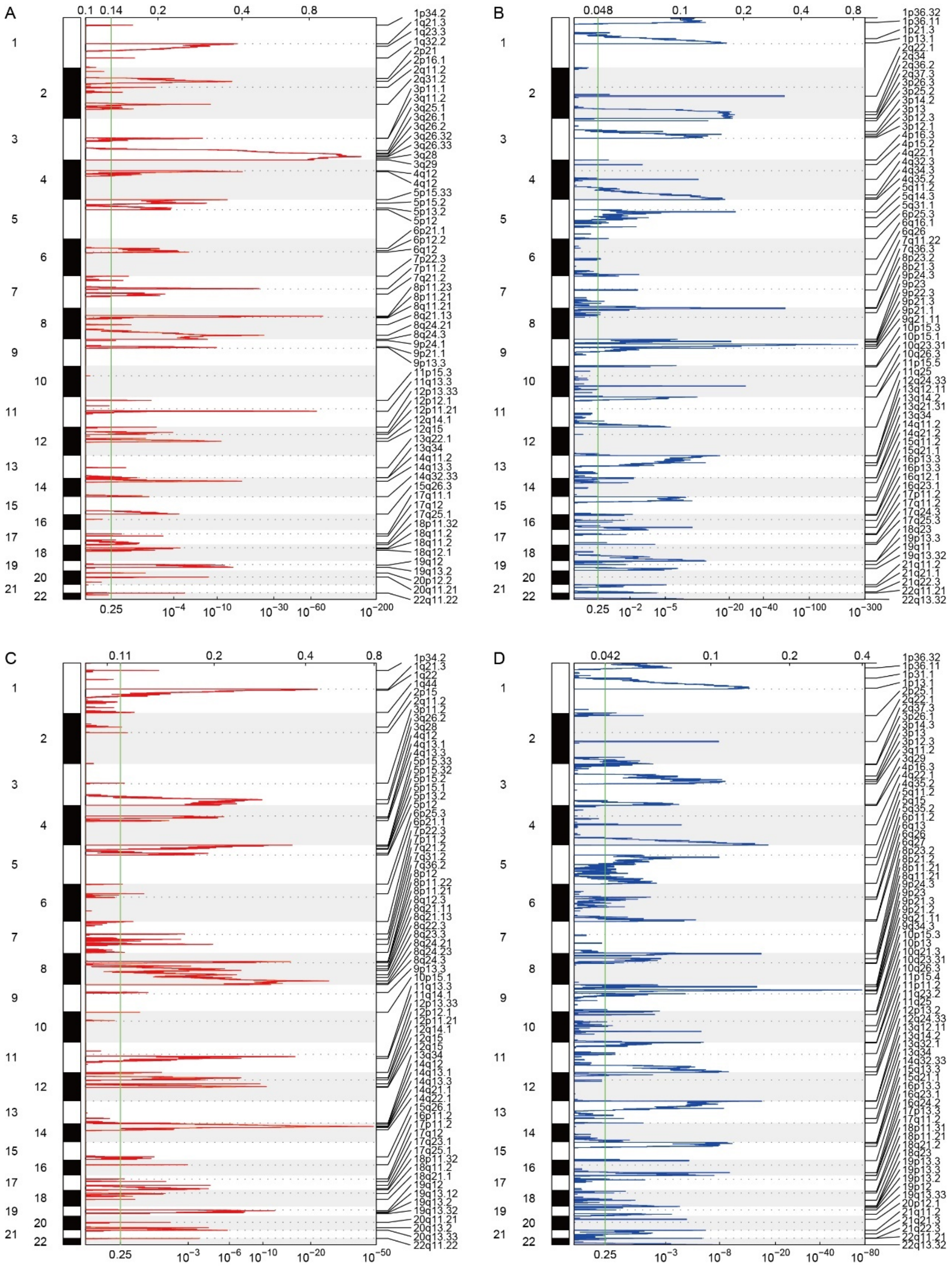

Supplementary Figure 1 Copy number variation analysis between the high and low DDR1 expression groups. **(A)**: Copy number amplifications in the high DDR1 expression group. **(B)**: Copy

number deletions in the high DDR1 expression group. **(C)**: Copy number amplifications in the low DDR1 expression group. **(D)**: Copy number deletions in the low DDR1 expression group.

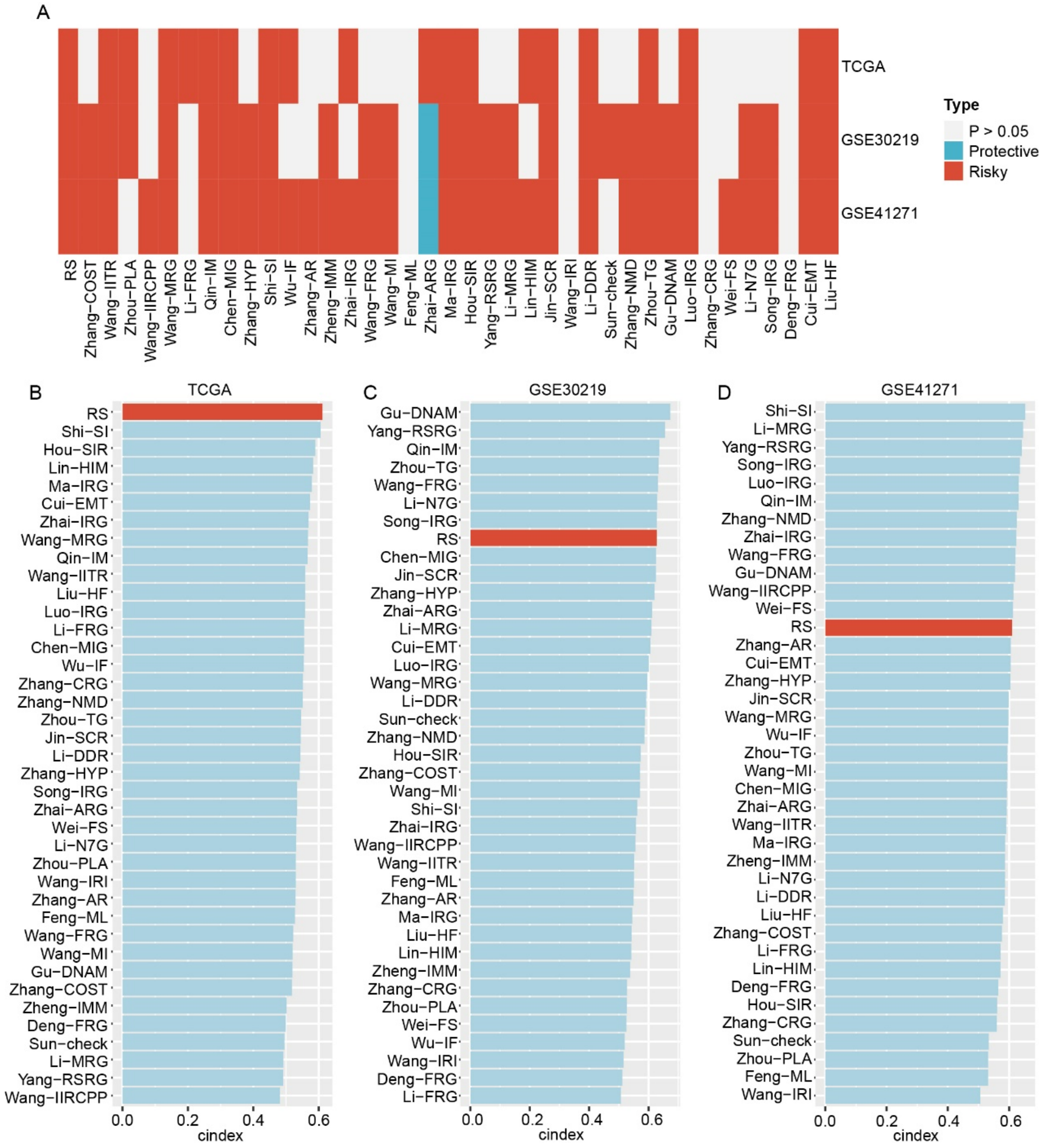

Supplementary Figure 2 Comparison of the prognostic model with other models. **(A)**: Univariate Cox analysis results. **(B)**: C-index values of the prognostic model and other models in the TCGA cohort. **(C)**: C-index values in the GSE30219 cohort. **(D)**: C-index values in the GSE41217 cohort

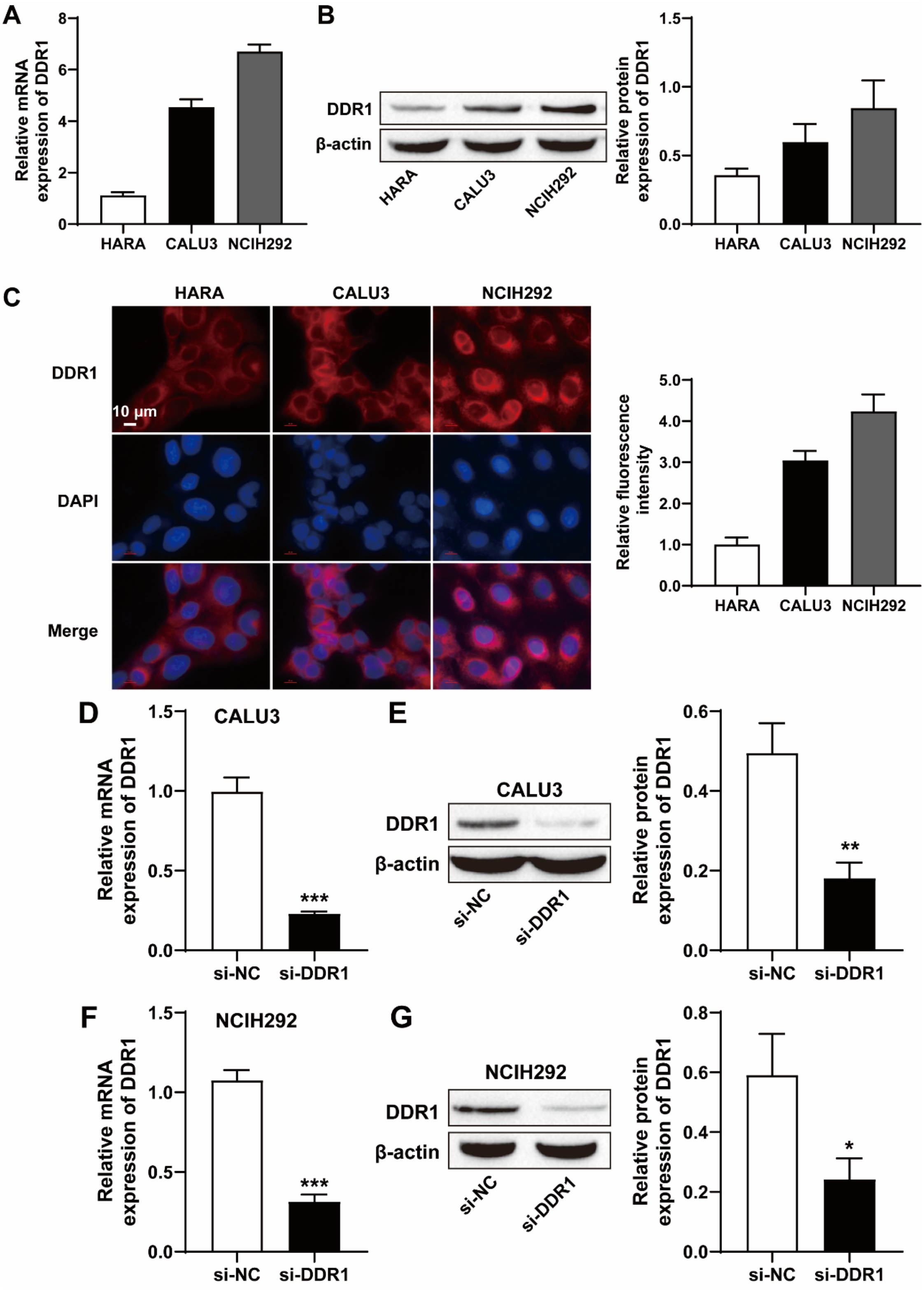

Supplementary Figure 3 RT-qPCR and western blotting validation of the knockdown efficiency of DDR1 siRNA. RT–qPCR **(A)**, western blotting **(B)**, and immunofluorescence **(C)**

confirmed the expression of DDR1 in the NSCLC cell lines HARA, CALU3, and NCIH292. RT–qPCR **(D and F)** and western blotting **(E and G)** verified the siRNA-mediated inhibition of DDR1 expression in CALU3 and NCIH292 cells. ***P<0.001, **P<0.01, *P<0.05. si, small interfering; NC, negative control.
